# Discounting Future Reward in an Uncertain World

**DOI:** 10.1037/dec0000219

**Published:** 2023-06-29

**Authors:** G. W. Story, Z. Kurth-Nelson, M. Moutoussis, K. Iigaya, G.-J. Will, T. U. Hauser, B. Blain, I. Vlaev, R. J. Dolan

**Affiliations:** 1 Division of Psychiatry, University College London; 2 Max Planck UCL Centre for Computational Psychiatry and Ageing Research, Institute of Neurology, University College London; 3 DeepMind, London, United Kingdom; 4 Wellcome Centre for Human Neuroimaging, Institute of Neurology, University College London; 5 Department of Psychiatry, Columbia University; 6 Division of Humanities and Social Sciences, California Institute of Technology; 7 Institute of Psychology, Leiden University; 8 Department of Psychiatry and Psychotherapy, University of Tubingen; 9 Department of Experimental Psychology, University College London; 10 Sorbonne Economics Centre, Sorbonne University; 11 Warwick Business School, University of Warwick

**Keywords:** discounting, impulsivity, uncertainty, risk, volatility

## Abstract

Humans discount delayed relative to more immediate reward. A plausible explanation is that impatience arises partly from uncertainty, or risk, implicit in delayed reward. Existing theories of discounting-as-risk focus on a probability that delayed reward will not materialize. By contrast, we examine how uncertainty in the magnitude of delayed reward contributes to delay discounting. We propose a model wherein reward is discounted proportional to the rate of random change in its magnitude across time, termed *volatility*. We find evidence to support this model across three experiments (total *N* = 158). First, using a task where participants chose when to sell products, whose price dynamics they previously learned, we show discounting increases in line with price volatility. Second, we show that this effect pertains over naturalistic delays of up to 4 months. Using functional magnetic resonance imaging, we observe a volatility-dependent decrease in functional hippocampal–prefrontal coupling during intertemporal choice. Third, we replicate these effects in a larger online sample, finding that volatility discounting within each task correlates with baseline discounting outside of the task. We conclude that delay discounting partly reflects time-dependent uncertainty about reward magnitude, that is volatility. Our model captures how discounting adapts to volatility, thereby partly accounting for individual differences in impatience. Our imaging findings suggest a putative mechanism whereby uncertainty reduces prospective simulation of future outcomes.

Humans and other animals exhibit impatience when offered reward (Berns et al., 2007; Frederick et al., 2002; Kalenscher & Pennartz, 2008; Loewenstein et al., 2003). Experiments show that the subjective value of reward decreases approximately in inverse proportion to its delay (Bickel et al., 2012; Green et al., 1994, 1999; Kirby et al., 1999; Madden et al., 2003). Specifically, people appear to *discount* the value of delayed reward following a hyperbolic function, such that the value of a reward of magnitude *X*, available after a delay *t* is given by the following equation:

$$V(X_{t})=\frac{X}{1+Kt^{s}}.$$ 1

Here *K* referred to as a *discount rate*, determines how steeply reward value decreases as it is delayed, with higher values of *K* generating greater impatience. *s* is an additional parameter corresponding to power law time perception (Rachlin, 2006). The wider relevance of delay discounting is supported by a correlation with impulsive or shortsighted real-world behavior, from substance misuse to undersaving for retirement (Bickel & Marsch, 2001; Bickel et al., 2014; Critchfield & Kollins, 2001; Daugherty & Brase, 2010; Epstein et al., 2014; Koffarnus et al., 2013; MacKillop et al., 2011; Meier & Sprenger, 2012; Snider et al., 2019).

An influential theory posits that delay discounting arises partly from uncertainty implicit in future reward (e.g., Andersen et al., 2008; Andreoni & Sprenger, 2012a; Harrison et al., 2005; Jones & Rachlin, 2009; Keren & Roelofsma, 1995; Kurth-Nelson et al., 2012; Luhmann et al., 2008; Noussair & Wu, 2006; Rachlin et al., 1991; Sozou, 1998; B. J. Weber & Chapman, 2005). Where the range of possible outcomes and their probabilities are precisely known, uncertainty over outcomes is termed “first-order uncertainty” (Bach et al., 2011), commonly conceptualized as “risk” (Von Neumann & Morgenstern, 1947). By contrast, uncertainty about the probability distribution of outcomes is referred to as “second-order uncertainty” (Bach et al., 2011; Knight, 1921), often conceptualized as “ambiguity” (Ellsberg, 1961). Here, for simplicity, we limit our discussion to first-order uncertainty, or risk.

Models of risk-sensitivity commonly assume that a preference for reward is affected by its variability (e.g., Bell, 1995; D’Acremont & Bossaerts, 2008; Kahneman & Tversky, 1979; Kroll et al., 1984; Sharpe, 1964; Symmonds et al., 2011; Weber et al., 2004). Furthermore, it has been proposed that delay discounting occurs because delayed reward entails greater risk than immediate reward (e.g., Jones & Rachlin, 2009; Rachlin et al., 1991; Sozou, 1998). However, rather than focusing on a variability associated with delayed reward, extant theories of discounting-as-risk have instead tended to emphasize a possibility that delayed reward will not be received. Such theories ascribe a probability per unit time that delayed reward will fail to materialize, termed a *hazard rate* (Friston et al., 2013; Jones & Rachlin, 2009; Kacelnik, 1997; Luhmann et al., 2008; Rachlin et al., 1991; Sozou, 1998; Yi & Landes, 2012). In support, delay discounting increases when people are faced with more hazardous life circumstances (Lahav et al., 2011; Sheffer et al., 2018) and is found to be higher both among people who are hopeless about the future (Pulcu et al., 2014), and among those who report reduced subjective survival probability (Chao et al., 2009). Furthermore, delay discounting correlates with a subjective probability that reward will not be paid out as promised (Patak & Reynolds, 2007; Reynolds et al., 2007; Takahashi et al., 2007).

Hazard rate models emphasize that delayed reward has a lower probability of being realized than immediate reward and hence has a lower expected (mean) value. By contrast, first-order uncertainty is greatest when all reward magnitudes are equally likely, which, in the case of reward *versus* no-reward, occurs when *p*(no-reward) = 0.5; see Rushworth & Behrens, 2008. Thus, if delay discounting is indeed sensitive to uncertainty about future reward then, even for instances where the expected (mean) value of future reward remains constant, a growing *variance* with time should lead to greater discounting. In other words, even where receipt of future reward is guaranteed, uncertainty about its magnitude may grow with delay. For example, owing to random changes in currency markets, the purchasing power of £100 1 year ahead is less predictable than it is today.

In support of this idea, delay discounting is higher in risk averse individuals (Eckel et al., 2005; Epper et al., 2011; Hayden & Platt, 2007; Leigh, 1986) and in those expressing a greater subjective intolerance of uncertainty (Luhmann et al., 2011). Finally, an observation that adding delay to choices between gambles reduces a bias toward riskless options (Noussair & Wu, 2006; B. J. Weber & Chapman, 2005) suggests that delay implicitly entails variability in reward. However, existing models of discounting make no claims about whether discounting is sensitive to future reward variance nor have previous approaches directly tested this experimentally. Previous work has examined uncertainty in the *timing* of future reward (Chesson & Viscusi, 2003; Dasgupta & Maskin, 2005); however, here we are concerned with magnitude uncertainty.

We address these issues here by formalizing first-order future uncertainty as *volatility*, the perceived rate of continuous random change in the reward environment, governing an increase in variance with delay (Figure 1a and b). We consider a simple model in which decision-makers discount future reward hyperbolically, with rate proportional to volatility. This model has a Bayesian interpretation as an optimal integration of future reward according to its magnitude uncertainty (see supplemental material; MacKay, 2003).[Fig fig1]

We adopt a modeling framework wherein agents have an internal, or generative model, of reward dynamics (cf. Behrens et al., 2007; Mathys et al., 2011). Within this framework, reward magnitude and its volatility are (subjective) parameters of the internal model. We assume that these parameters are learned and therefore come to reflect both an agent’s preferences and the objective dynamics of the environment. Thus, magnitude uncertainty might entail either variance in the objective size of rewards (e.g., the value of a stock, or the weight of corn produced per hectare of land), or in the *subjective* value of reward (e.g., appetite for food, fashion tastes). Here, we manipulate volatility in objective reward magnitude, though we consider the very same model could be applied to volatility in subjective value.

## A Volatility Discounting Model

We consider a situation wherein a decision-maker is promised a reward, with an expected magnitude, if redeemed immediately, of *R*_0_ (e.g., a £10 voucher for a local deli, which is expected to buy a kilogram of food). However, the reward is only available after a delay, *t* (e.g., the voucher is redeemable after a month). The decision-maker believes the magnitude of the reward might change during the delay (e.g., the price of food at the deli may fall or rise, meaning that the voucher will buy either more or less food).

Specifically, we assume that the decision-maker is equipped with a generative model wherein expected future reward magnitude, *R*_*t*_ > 0, evolves based on a random walk, with *volatility*, σ^2^, such that:

$$R_{t}=R_{t-1}+\varepsilon_{t},\;\varepsilon_{t}∼N(0,\;\sigma^{2}).$$ 2

We also consider an additional “emission noise” in the observed rewards, *r*_*t*_, which does not vary with time (e.g., portion sizes vary slightly from day-to-day), and has standard deviation, ϑ:

$$r_{t}=R_{t}+\varepsilon_{0},\;\varepsilon_{0}∼N(0,\;\vartheta^{2}).$$ 3

A key intuition here is that a latent quantity, *R*_*t*_, governs reward magnitude and varies over time. However, the reward realized at a given time step, *r*_*t*_, is not precisely equal to *R*_*t*_, but instead it is drawn from a Gaussian distribution with a mean of *R*_*t*_.

Since there is no drift in the random walk and no bias in the emission noise, future reward has the same expected (mean) magnitude as if it were consumed immediately (e.g., one can expect, on average, a £10 voucher will buy the same amount of food next month as it would buy today):

$$E[r_{t}]=E[R_{t}]=R_{0}.$$ 4

However, by the additive property of Gaussian random variables, the variance of future reward grows linearly with delay, reflecting an accumulation of random changes over time:

$$var[r_{t}]=t\sigma^{2}+\vartheta^{2}.$$ 5

Thus volatility, σ^2^, determines how quickly uncertainty in reward magnitude grows with delay (e.g., changeability in food prices). The above definition of volatility follows previous learning models (Behrens et al., 2007; Mathys et al., 2011); formally similar definitions occur in financial modeling (e.g., Black & Scholes, 1973; Sharpe, 1964).

We posit that, given estimates of σ^2^ and ϑ^2^, the decision-maker values reward in inverse proportion to its variance:

$$V(r_{t})=\frac{E\left[r_{t}\right]}{var\left[r_{t}\right]}.$$ 6

Here, rewards whose value is more precisely known contribute more to a long-run estimate of future value; distant rewards are more uncertain, and therefore receive less weight (MacKay, 2003).

We envisage σ^2^ and ϑ^2^ as parameters within the decision-maker’s internal model of reward dynamics. Here we assume that the volatility of the reward environment is static, and known to the decision-maker; we therefore fix σ^2^ to the objective, external volatility. However, in Equation 6, the value of reward tends to infinity as its variance tends to zero. To address this, we define the emission noise parameter, ϑ^2^, as a sum of internal and external sources of noise:

$$\vartheta^{2}=\eta^{2}+\theta^{2}.$$ 7

where η^2^ denotes the objective, external noise, and θ^2^ denotes a degree of irreducible *internal* uncertainty associated with all reward, even a nominally certain reward. As described below, this formalism allows θ^2^ to capture individual differences in sensitivity to variance in objective reward magnitude.

To derive a discount function, we consider a choice between an immediate reward, *E*[*r*_*t*_] = *x*, and a larger delayed reward, *E*[*r*_*t*_] = *X*. At indifference, by Equation 6:

$$\frac{x}{X}=\frac{var\left[r_{0}\right]}{var\left[r_{t}\right]}.$$ 8

Thus, delayed reward is discounted according to its uncertainty, relative to that of immediate reward. For a case, where immediate reward is nominally certain:

$$\frac{x}{X}=\frac{\theta^{2}}{\theta^{2}+\eta^{2}+t\sigma^{2}}.$$ 9

Dividing by θ^2^ and substituting *m* = 1/θ^2^ gives the following equation:

$$\frac{x}{X}=\frac{1}{1+m(\eta^{2}+t\sigma^{2})}.$$ 10

This arrangement thus yields hyperbolic discounting of risky reward according to its objective variance, with rate *m*. Where *t* = 0, *m* captures individual differences in *risk aversion*, with the variance of a risky prospect given by η^2^.

In fitting the model, we also allow for a possibility of nonlinear time perception, whereby uncertainty increases as a function of subjective time:

$$\frac{x}{X}=\frac{1}{1+m(\eta^{2}+\sigma^{2}t^{s})}.$$ 11

**Figure 1 fig1:**
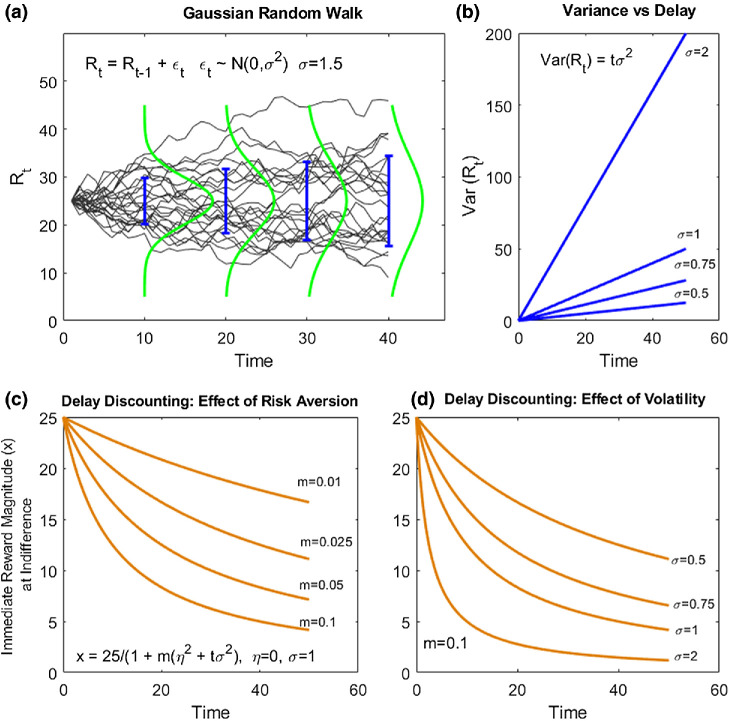
A Volatility Discounting Model *Note*. (a) Samples of reward magnitude drawn from a Gaussian random walk. Solid blue bars represent 1 *SD* above and below the mean at each timepoint. Green lines schematically represent Gaussian probability densities at the same timepoints. (b) Variance in expected reward magnitude grows linearly with delay with a slope given by volatility. (c) Immediate reward magnitude, *x*, at indifference, is plotted as a function of time delay for different levels of risk aversion (left panel) and volatility (right panel). Rewards are hyperbolically discounted, with discount rate given by an interaction between risk aversion *m* and volatility, σ^2^. Here, for simplicity, we assume η = 0, *s* = 1. See the online article for the color version of this figure.

This arrangement is commensurate with existing models of hyperbolic discounting (Rachlin, 2006). Where objective time-independent risk is negligible (η^2^ = 0), Equation 11 obtains hyperbolic delay discounting (as shown in Equation 1), with rate proportional to volatility, *K* = *m*σ^2^, and where individual differences in “volatility discounting” are captured by *m* (shown in Figure 1c).

Gabaix and Laibson (2017) derive a closely related model by considering a decision-maker’s internal uncertainty associated with future reward. In the resulting model, agents attempt to mentally simulate the future and combine these noisy signals with their prior expectations to generate posterior beliefs. By analogy to volatility, noise in the sampling process accrues linearly with delay. The authors show that this arrangement results in hyperbolic discounting, with a rate given by the precision of future simulations relative to prior expectations. A corollary of this arrangement is that at long delays, where the value of future reward is imprecisely known, average value estimates return to prior expectations.

Gershman and Bhui (2020) extend the above framework by postulating that more precise future simulation demands mental effort. The authors show how this model can account for a well-known finding that people are more patient for larger rewards, by proposing that increased simulation effort is deemed more worthwhile for larger reward. Their model also accounts for a finding that choices are more variable for smaller rewards, an effect that is found to diminish for more uncertain rewards. Here by contrast we experimentally vary volatility in *external* reward. The present study therefore links Bayesian approaches to discounting with extant notions of discounting-as-risk.

## Neural Correlates of Volatility Discounting

A further outstanding question is how uncertainty-dependent valuation of future reward is implemented in the brain. A process model, we consider, is that uncertainty reduces the extent to which future outcomes are incorporated into a person’s model of their future situation (Gershman & Daw, 2017; Kurth-Nelson et al., 2012; Schacter et al., 2008). Such high-level models are supported by medial temporal lobe (MTL) and related structures, in particular, the hippocampus (HC; Addis et al., 2007; Hassabis et al., 2007; Johnson & Redish, 2007; Peters & Büchel, 2010; Schacter et al., 2008; Tsao et al., 2018). Important here are observations that encouraging imagination of delayed reward, by embedding rewards within future episodes of a person’s life (e.g., an upcoming birthday), decreases delay discounting (Daniel et al., 2015; Peters & Büchel, 2010) and increases connectivity between MTL and prefrontal regions encoding discounting value (Peters & Büchel, 2010). However, if future rewards are more variable then representing the ensuing range of future scenarios is more cognitively demanding (Gershman & Bhui, 2020). We suggest therefore that, when faced with uncertain future reward, rather than expend cognitive resource, people engage simpler value representations that are less enriched by episodic forecasts. This would predict that MTL regions should then participate less in the evaluation of more volatile delayed rewards, and in functional magnetic resonance imaging (fMRI), this would predict a decreased correlation between MTL activity and discounted value, and/or a weakening of MTL connectivity with regions representing discounted value (Kable & Glimcher, 2007; McClure et al., 2004, 2007).

## Summary of Experiments

We test the above predictions across three experiments (total *N* = 158); wherein, we manipulate reward volatility in a combined learning and intertemporal choice (ITC) task. In Experiment 2, a subset of participants performed the task whilst undergoing fMRI, to probe a relationship between uncertainty-dependent valuation of future reward and MTL activity. Notably, in reinforcement-learning models, discounting of past reward is determined by a learning rate, where a high learning rate entails steeper discounting of past rewards and faster value updates (Daw et al., 2005, 2006; Dolan & Dayan, 2013; Mathys et al., 2011; Rescorla & Wagner, 1972; Sutton & Barto, 1998; Wilson et al., 2010). As predicted by optimality, learning rates in humans increase when reward contingencies are more changeable, or volatile (Behrens et al., 2007; Iigaya, 2016; McGuire et al., 2014; Nassar et al., 2010, 2012). Both learning rate and discount rate ought therefore to increase when reward dynamics become more changeable. Furthermore, if learning rate and discount rate both depend on perceived volatility, then the two parameters might be correlated across participants. We also examine these hitherto untested predictions.

## Experiment 1

**Figure 2 fig2:**
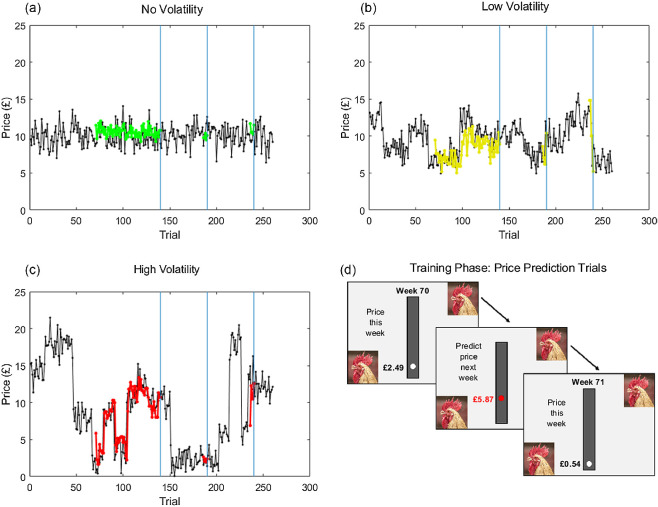
Design of Experiment 1: Farming Futures Task *Note*. Participants tracked prices of three agricultural products, where a single trial corresponded to a “week.” For one product (a), no volatility, the underlying market price was constant, with added Gaussian emission noise. For another two products, the market price underwent shifts across time, with the same emission noise. For a low volatility product (b) shifts in the market price were small, while for a high volatility product (c), shifts were more extreme. (d) After observing prices over several trials, participants were asked to predict upcoming prices 1 week ahead. Within each block, participants performed three phases of observation and prediction: The first consisted of 70 observation trials followed by 70 prediction trials, while the subsequent two phases each consisted of 45 observation trials and 5 prediction trials. (Example predictions from one participant shown in color in Panels a–c). The time series was paused at three points (vertical blue lines), where participants predicted market prices further into the future. They were subsequently asked to indicate the lowest price they would accept to sell the product on the market after a delay (not shown), providing an estimate of the discount factor at each delay. See the online article for the color version of this figure.

In Experiment 1, participants were briefed to imagine that they owned a farming business, selling produce to the highest bidder in a marketplace. Participants learned how the prices of three different products (wheat, chicken, and beans) evolved week-by-week, where a week corresponded to a trial of the experiment (Figure 2). The three products had different levels of volatility in price evolution. Participants subsequently made ITCs about when to sell each product, either immediately for a guaranteed price or in the marketplace following a delay.[Fig fig2]

### Method

#### Ethics Statement

All participants gave full informed consent before taking part in the study. The study procedures received approval from the UCL Research Ethics Committee (3450/002) and were carried out in accordance with these guidelines.

#### Data and Code Availability

Behavioral data supporting the findings of this study are publicly available online in a third-party repository: https://doi.org/10.5061/dryad.47d7wm3k2 (G. Story, 2023). Computer codes that support the findings of this study are available from the corresponding author upon reasonable request.

#### Participant Recruitment and Sample Size

This experiment was designed as a pilot, and thereby focused on testing for larger, within participant, effects. Participants were recruited from the UCL Institute of Cognitive Neuroscience subject database. Twenty participants (mean age 27.4 years, *SD* 6.9 years; nine female) completed the experiment.

#### Baseline Discounting

Prior to the main task, we elicited discount functions for riskless quantities of money. Participants were required to indicate the smallest immediate monetary reward, termed their indifference amount, that they would be willing to accept instead of a larger stated quantity of money (£8, £9, £11, or £12) to be received at a specified delay (1, 2, 4, 26, or 52 weeks). Each delay was presented twice for each larger reward amount, creating 40 choices in total. One choice was selected to be paid for real, at the stated delay, in postdated Amazon vouchers. To achieve this in an incentive-compatible manner, for the selected choice, we randomly selected an immediate reward from a uniform distribution between £0 and the magnitude of the larger reward (e.g., £12); if this amount was below or equal to the participant’s stated indifference point, they received the delayed reward, if above the indifference point they received the randomly drawn immediate reward. Participants were fully briefed on this procedure. We fitted a two-parameter hyperbolic model of the form shown in Equation 1 to participants’ indifference points (Rachlin, 2006).

#### Learning Price Dynamics

During the task, participants observed and predicted the price of each product, displayed on a linear scale ranging from £0 to £25, as it evolved over the course of 240 trials. Each trial of the experiment was described as a “week.” After passively observing prices over several “weeks” (trials), participants were asked to predict upcoming prices 1 week ahead; the task therefore involved both observational and instrumental learning (Figure 2a–c). Participants were instructed about two sources of variability in prices: Gaussian emission noise, applying equally to all products, which we described as “variability in bidding,” and changes in the underlying “market price.” For one of the three products (“no volatility”), the market price was held constant; the market price of the other two products (“low volatility” and “high volatility”) underwent random changes across time, with the same Gaussian emission noise. We used two predefined sequences of outcomes for each product; participants were then allocated at random to one of the two sequences. We estimated learning rates for the three products separately by fitting a Rescorla–Wagner learning model (see supplemental material; Rescorla & Wagner, 1972) to participants’ price predictions from the first block of 70 prediction trials (shown in Figure 2a–c).

#### ITC Procedure

At three points during each block, participants were asked to predict the market price further into the future, at delays of 1, 4, 7, 12, or 18 weeks. Participants subsequently chose when to sell the product, either immediately for a fixed price (*x*), or on the market after a stated delay (1, 4, 7, 12, or 18 weeks). Specifically, they were asked to indicate the smallest fixed price that would just tempt them away from selling on the market. Participants were informed that the future price would evolve according to the same process they had previously observed and was also subject to the same Gaussian emission noise. By contrast, the immediate price was fixed, with no objective risk.

Participants were informed that, after the experiment, we would select one of their choices to be paid out for real. To realize this in an incentive-compatible manner, for the selected choice, we randomly selected an immediate fixed price from a uniform distribution between £0 and £25; if this amount was below the participant’s stated indifference point, they received the simulated future market price for the product as a bonus payment. If the selected price was above the participant’s indifference point, they received the randomly drawn fixed price. All bonus payments were made on the same day, at the end of the experiment.

#### Statistical Analyses

We used a Bayesian hierarchical mixed-effects model fitting routine, the details of which have been described previously (Huys et al., 2011, 2012). Models were compared using the integrated Bayesian information criterion (BIC*i*), which approximates the model evidence (Huys et al., 2011, 2012). We also computed exceedance probabilities (φ), which estimate the likelihood of a given model outperforming the alternatives, given the model evidence for each across participants (Stephan et al., 2009).

We fitted participants’ reported indifference points with a volatility discounting model. The model assumed two independent contributions to discounting: a baseline component due to effects other than volatility, with rate *K*, (see Equation 1) and an additional component due to volatility (see Equation 11):

$$x=\frac{X}{1+Kt^{s}}\cdot \frac{1}{1+m(\eta^{2}+\sigma^{2}t^{s})}+c.$$ 12

Here *x* represents the participant’s immediate price at indifference, while *X* represents the expected future market price. We tested alternative models in which *X* was given by either (a) each participant’s individual estimate of the future price at the relevant delay, or (b) the mean of this estimate across participants. σ^2^ represents objective volatility, and η^2^ objective emission noise. *m* is a participant-specific risk aversion parameter. *c* is a bias term that does not vary with condition and is set to zero for immediate options. *s* is an additional parameter corresponding to power law time perception (Rachlin, 2006). Using nonlinear optimization in MATLAB (Mathworks, Provo), with a Gaussian likelihood function, parameters were sought that minimized differences between reported indifference amounts and those predicted by the model.

We first fitted hyperbolic curves to each product separately, omitting the volatility discounting term (setting *m* = 0), and testing for an effect of volatility on log *K* using linear mixed-effects regression. The contribution of each participants’ data to this analysis was weighted by the reliability of their log *K* estimates (see Huys et al., 2012). We went on to fit the full model to the three products jointly, with differences between products parameterized by *m*. Finally, we tested a different class of model wherein risk preference is accounted for by concave utility over reward magnitude (see supplemental material).

### Results

#### In-Task Discount Rate Increased With Volatility

**Figure 3 fig3:**
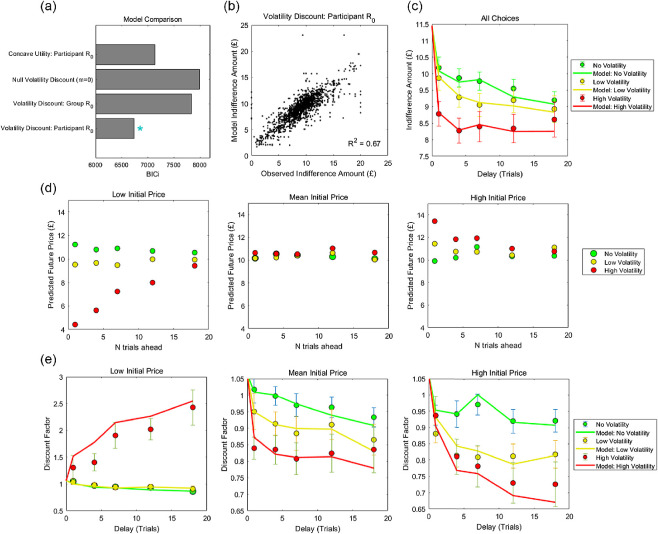
Model Fits, Price Forecasts, and Discount Factors in Experiment 1 *Note*. (a) Model comparison; blue asterisk indicates the best fitting volatility discounting model. (b) Observed indifference amounts are plotted against indifference amounts predicted by the best fitting model. (c) Mean indifference amounts (immediate selling prices) across all choices are plotted as a function of delay, separated by product (*N* = 20 participants). Solid lines show the fits of a volatility discounting model, where subjective forecasted future prices are hyperbolically discounted according to their variance. (d) Mean price forecasts for the three products separated by initial market price for the volatile products. (e) To illustrate effects of initial price on delay discounting, indifference amounts are transformed to discount factors (immediate price/future price), using the *initial market price* as an estimate of the future price. Indifference amounts predicted by the model are also divided by the initial market price, and overlaid with the observed data. When the price of the high volatility product was expected to rise, participants chose to defer selling the product (left panel). By contrast, participants were more likely to sell the high volatility product immediately when its price was expected to fall (right panel). When the expected future price was constant (central panel), discounting increased with increasing volatility. Throughout, error bars indicate one standard error. BIC*i* = integrated Bayesian information criterion. See the online article for the color version of this figure.

As shown in Figure 3a, a volatility discounting model in which future reward magnitude was estimated from participants’ individual price predictions outperformed a version based on mean price predictions across participants (ΔBIC*i* = 1,104; higher model evidence in 16/20 participants; φ = 0.998). This model also outperformed a null model in which *m* = 0 (ΔBIC*i* = 1,252; higher model evidence in 16/20 participants; φ = 0.999), and an alternative model based on concave utility (ΔBIC*i* = 400; higher model evidence in 16/20 participants; φ = 0.999), supporting an effect of volatility discounting.[Fig fig3]

As shown in Figure 3b and c, a volatility discounting model (Equation 12) provided a good fit to participants’ choices. Figure 3c shows observed indifference amounts for the three products, averaged across choices and participants, together with those predicted by the model. Discounting increased in proportion to volatility, linear mixed effects on log *K* fitted to each product separately: β_condition_ = 0.19, *t*(58) = 2.38, *p* = .020.

Participants’ future price predictions for the three products are shown in Figure 3d. For the purposes of illustrating effects of initial price on delay discounting, we transformed indifference amounts to discount factors (immediate price/future price), using the *initial market price* as an estimate of the future price. Indifference amounts predicted by the model were transformed in the same way, overlaid with the observed discount factors in Figure 3e.

When the initial price was close to the long-run mean, participants correctly predicted zero net growth in price, and discounting increased with increasing volatility. For the high volatility product, when the current price was below average, participants expected the future price to increase across time, and accordingly showed a preference to defer reward. For the low volatility product, when the current price was below average, expected price increases were more subtle, and discount factors predominantly reflected a small effect of volatility. By contrast, when the initial price was above average, participants expected the price of the high and low volatility items to fall and accordingly were more likely to prefer the immediate reward. These effects reflect the experimental design, wherein price evolution was bounded between £0 and £25, leading participants to expect that eccentric prices would tend to return toward the long-run average. The effects are captured by the model, which is furnished with participants’ subjective estimates of future market prices.

#### Baseline Discount Rate Correlated With In-Task Volatility Discounting

Importantly, volatility discounting within the task, governed by parameter *m*, showed a significant positive correlation with baseline discounting (Spearman ρ = 0.49, *p* = .049; *N* = 17; three participants who answered £0 in response to all baseline questions were excluded from this analysis). That is, people who showed greater discounting of uncertainty within the task tended to show steeper discounting of money outside of the task.

#### Learning Rate Increased With Volatility

As previously reported (Behrens et al., 2007; Lee et al., 2020), Rescorla–Wagner learning rates increased with increasing volatility, linear mixed effects on α: β_condition_ = 0.88, *t*(58) = 6.61, *p* < .001; no volatility: mean α = 0.39, *SD* = 0.22, low volatility, mean α = 0.55, *SD* = 0.17, high volatility mean α = 0.75, *SD* = 0.11. However, in-task discounting showed no significant correlation with learning rate across participants in any of three conditions (log *K* vs. α; no volatility: ρ = 0.41, *p* = .076; low volatility: ρ = −0.12, *p* = .612; high volatility: ρ = −0.28, *p* = .232; supplemental Figure S1).

### Summary

In summary, in this pilot experiment, we found that within-task delay discounting increased in line with reward volatility. Furthermore, people who showed greater volatility-dependent increases in discounting within the task tended to show steeper discounting of money across real delays at baseline. This finding supports an hypothesis that discounting of time-dependent uncertainty contributes to individual differences in delay discounting.

## Experiment 2

Experiment 2 tested whether the effects observed in Experiment 1 replicated in a larger sample and also probed neural correlates of volatility discounting. Here, to test whether effects of volatility extend to timescales used in conventional discounting tasks, we superimposed the timescale of the task onto longer delays. Specifically, one actual ITC was selected to be paid out at the stated delay, in the order of weeks. To further test the veridicality of the model, we measured risk aversion outside the main task, and elicited participants’ subjective estimates of future uncertainty within-task.

### Method

#### Ethics Statement

All participants gave full informed consent before taking part in the study. The study procedures received approval from the UCL Research Ethics Committee (3450/002) and were carried out in accordance with these guidelines.

#### Data and Code Availability

Behavioral data supporting the findings of this study are publicly available online in a third-party repository: https://doi.org/10.5061/dryad.47d7wm3k2 (G. Story, 2023). Computer codes and imaging data that support the findings of this study are available from the corresponding author upon reasonable request.

#### Learning Phase

**Figure 4 fig4:**
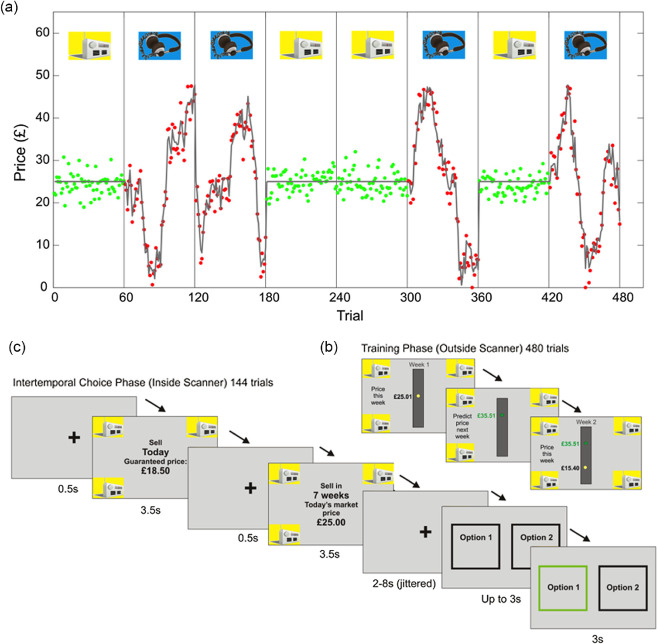
Design of Experiment 2: Online Marketplace Task *Note*. (a) Participants first observed prices of two products to be sold in an imaginary online marketplace, where one trial corresponded to 1 week. For one of the two products, the market price (solid gray line) was constant at £25, with added Gaussian emission noise. For the other product, the market price changed as a Gaussian random walk (volatility σ = 3.5), with the same emission noise (standard deviationη = 2). Sample inputs from one participant are shown. (b) Participants predicted prices 1 week ahead. (c) Subsequently, inside the fMRI scanner, participants chose between selling each product today for a guaranteed price, or after a delay in the simulated online marketplace. This differs from a standard delay discounting task because the uncertainty of the future payoff in the volatile condition grows with delay. See the online article for the color version of this figure.

Participants learned price dynamics according to a similar procedure as described for Experiment 1 (see supplemental material and Figure 4a and b). Here only two products were used, to simplify the neuroimaging analysis. For one of the two products (“stable”), the market price was held constant at £25, and participants were explicitly informed about this; the market price of the other product (“volatile”) evolved according to a Gaussian random walk, with zero mean drift and volatility σ = 3.5, upper bounded at £50 and lower bounded at £0 (Figure 4a). We used two predefined sequences of outcomes sampled from a random walk with these properties; participants were then allocated at random to one of the two sequences. To estimate the optimal learning rate for the volatile product, we fitted a Rescorla–Wagner model so as to minimize differences between observed price inputs and those predicted by the model. We did this for the specific stimulus sequence observed by each participant, and averaged the estimates.[Fig fig4]

#### ITC Phase

After observing price evolution for both products, participants entered a separate ITC phase of the experiment. Here, participants made a series of binary choices about when to sell each product, either immediately for a guaranteed (i.e., riskless) price (less than £25), or in the marketplace after a stated delay (0, 1, 4, 17 weeks) from a starting price of £25 (Figure 4c). We included a delay of 0 week to allow the intercept of the discounting curve to be reliably estimated. We selected a set of guaranteed immediate prices that allowed a plausible range of discount factors to be estimated (the full choice set, with equivalent *K* values at indifference, is shown in supplemental Table 4).

Participants were informed that one of their choices would be selected and realized after the experiment and, depending on their actual choice, participants would be paid either the guaranteed amount on the day of the experiment (if they opted for the immediate choice), or the simulated future price of the product *at the stated number of weeks in the future* (if they opted for the delayed choice). Here, since delays were real, rather than embedded within the timescale of the task, we expected a significant degree of discounting even in the stable condition, due to the influence of effects outside of the task. Modeling analyses followed equivalent procedures as described for Experiment 1: we fitted log *K* separately for the two products and also fitted volatility discounting models. We also tested a model wherein risk preference is accounted for by concave utility over reward magnitude.

#### Participant Recruitment, Sample Size, and Power Calculation

We conducted a behavioral pilot experiment with 11 participants, using the above design. In this pilot experiment, we observed an effect size of *d* = 0.76, based on the mean difference in log *K* between stable and volatile products, suggesting that a medium effect size was a plausible assumption. Sample size for the imaging experiment was therefore determined so as to achieve at least 80% power to detect a medium effect size (Cohen’s *d* = 0.5), based on a paired *t*-test, indicating a required sample size of 34 participants. We aimed to recruit at least 34 participants for the main experiment, in addition to the pilot sample. Thirty-six participants were recruited from the UCL Institute of Cognitive Neuroscience subject database, and underwent MRI scanning. Including the behavioral pilot, a total of 47 participants (mean age 28.0 years, *SD* 8.4 years, 32 female) completed the experiment.

#### fMRI Methods

Imaging methods are described in online supplemental material.

#### Baseline Risk Aversion

Before participants were introduced to the market behavior, we measured their risk preferences for lotteries to be paid out on the same day. Each lottery had prices drawn from a Gaussian distribution with mean £25, and one of four standard deviations (ranging from £5 to £13). For each lottery participants observed 36 outcomes drawn from the relevant distribution, before making a series of choices between receiving a guaranteed amount (between £11 and £25) or accepting a one-off play of the lottery. We fitted participants’ risk choices with a hyperbolic risk discounting model of the form shown in Equation 12, with *t* = 0, setting η^2^ to the objective variance of the lottery. We also tested a standard additive mean–variance model (Bell, 1995; D’Acremont & Bossaerts, 2008; Kroll et al., 1984; Symmonds et al., 2011; Weber et al., 2004; see supplemental material).

#### Subjective Future Uncertainty

Following the learning phase, we elicited participants’ predictions about each product’s future price, for a scenario in which the current market price was stated to be £25. Using a graphical interface, participants were also asked to indicate the lower and upper bounds of an interval in which they were 90% certain the future price would lie, described as “highest and lowest reasonable estimates” (for similar estimation procedures see Cesarini et al., 2006; Delavande & Rohwedder, 2008; O’Connor, 1989). We fitted a model to participants’ confidence intervals based on the true generative process, that is a Gaussian random walk (see supplemental material), to derive a subjective estimate of σ^2^ for each participant in each condition, which we termed “subjective future uncertainty” ($\text{SF}U\overset{\text{def}}{=}{\hat{\sigma }}^{2}$).

#### Statistical Analyses

As shown in Figure 1, the volatility discounting model predicts that a component of discounting is proportional to an interaction between future uncertainty (σ^2^) and risk aversion (*m*). To test this, we examined a relationship between log *K* and an (SFU × *m*) interaction, where *m* is measured from risk choices outside of the discounting task. The interaction term denotes the “subjective cost of future uncertainty.” We tested for an effect on log *K* of a subjective cost of future uncertainty in stable and volatile conditions separately, using simple linear regression. Here, the contribution of each participants’ data was weighted by the reliability of their log *K* estimates (see Huys et al., 2012), where log *K* was estimated separately for the two conditions (setting *m* = 0 when fitting discounting choices).

We also tested whether an effect of condition on discounting was greater amongst participants who showed a greater *increase* in the subjective cost of uncertainty between conditions. To do so, we calculated, for each participant, the change in SFU between conditions: dSFU = SFU_volatile_−SFU_stable_. We then implemented a mixed-effects linear regression on log *K*, with *m*, dSFU, condition, (Condition × dSFU), (Condition × *m*), and (Condition × dSFU × *m*) as predictor variables, where *condition* is coded as a within-subject dummy variable. We hypothesized that participants who were (a) risk averse and (b) showed a greater subjective increase in uncertainty between conditions would be more sensitive to an effect of condition, manifest in a significant three-way (Condition × dSFU × *m*) interaction.

### Results

#### Baseline Risk Aversion

A hyperbolic risk discounting model substantially outperformed a standard mean–variance model in accounting for baseline risk preferences (higher model evidence in 39/45 participants; φ > 0.999). Participants were on average risk averse (mean log *m* = −7.44, 95% CI [−7.82, −7.06]) and also showed a bias away from choice of the risky option (mean *c* = 3.43, 95% CI [4.00, 2.86]).

#### Subjective Future Uncertainty Increased With Volatility

**Figure 5 fig5:**
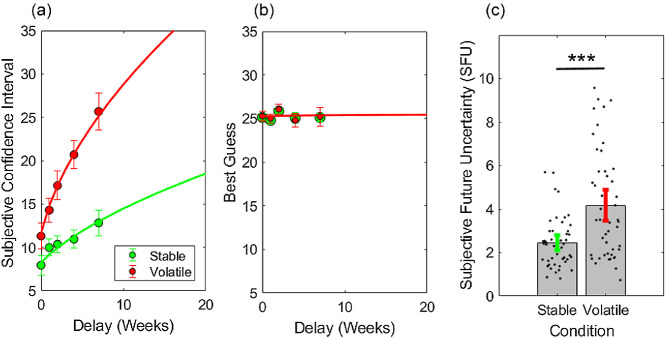
Subjective Future Uncertainty and Price Forecasts in Experiment 2 *Note*. (a) Mean width of subjective confidence intervals on future prices. Participants accurately predicted that uncertainty grows as a function of delay in the volatile condition (red). Solid lines show the fit of a random walk model. (b) Mean “best guesses” about future prices. Participants accurately predicted that expected prices are constant across delay. Solid lines show the fit of log growth curves. *N* = 47 for all analyses shown. Error bars indicate one standard error. (c) Subjective future uncertainty (SFU), that is subjective volatility $\hat{\sigma }$ derived from growth in confidence intervals across delay. Filled gray bars show group means, solid green and red bars 95% CIs; black dots show individual parameter estimates. See the online article for the color version of this figure. *** *p* < .001.

As shown in Figure 5a, subjective confidence intervals increased with delay. On average, in the volatile condition participants accurately predicted that the expected future price would remain £25 (Figure 5b; mean slope of future predictions, β = 0.03, 95% CI of β [−0.38, 0.44]). Consistent with an effect of volatility, SFU was significantly greater in the volatile than in the stable condition, paired *t*(46) = 8.7, two-tailed *p* < .0001, *d* = 1.27; Figure 5a and c. In the volatile condition, participants tended to slightly overestimate the true volatility (group mean $\hat{\sigma }=4.17$, true σ = 3.50, 95% CI of $\hat{\sigma }$ [3.44, 4.90]). Notably there was a shallow positive slope even in the stable condition, in keeping with a prior belief that uncertainty grows with delay (group mean $\hat{\sigma }=2.08$, true σ = 0, 95% CI of $\hat{\sigma }$ [2.11, 2.80]).[Fig fig5]

#### Delay Discounting Increased With Volatility

**Figure 6 fig6:**
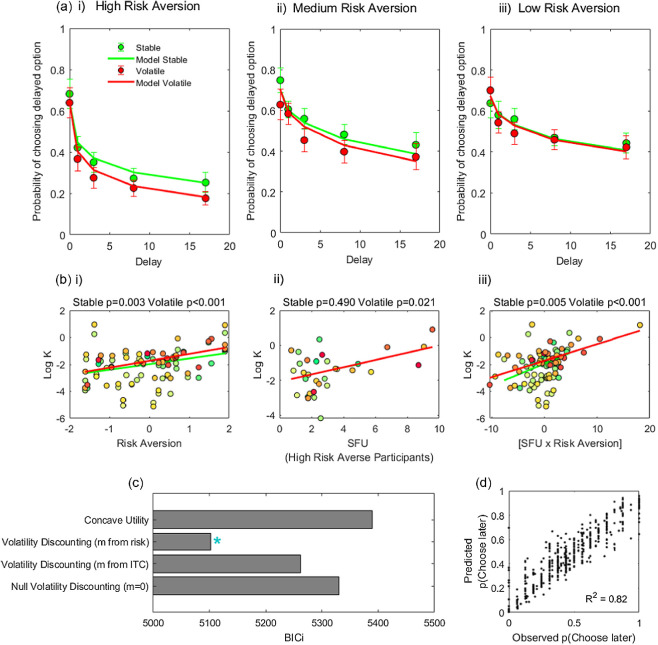
Delay Discounting as a Function of Risk Aversion and Future Uncertainty in Experiment 2 *Note*. (a) Mean discount functions for stable (green) and volatile conditions (red) for participants separated by risk aversion, *N* = 15 per tertile. Low risk aversion: log (*m*) ≤ −8.06; medium: −8.06 < log(*m*) ≤ −4.75; high: log (*m*) > −4.75. Error bars represent 1 *SE*. Solid lines represent fits of a volatility discounting Model to both conditions together, with risk aversion, *m*, estimated from risk choices outside of the ITC task. (b) Discounting depends on the subjective cost of future uncertainty. Log *K* fitted directly to volatile (red) and stable (green) conditions is plotted as a function of risk aversion (*m*, *Z*-scored), SFU (shown here for high risk averse participants, *N* = 15) and an (SFU × Risk Aversion) interaction. Color intensity reflects the contribution of each data point to the weighted least squares regression. P-values are for each condition and regressor separately. (c) Model comparison; blue asterisk indicates the best fitting volatility discounting model, with risk aversion, *m*, estimated from risk choices outside of the ITC task. ITC = intertemporal choice. (d) Observed choice proportions are plotted against proportions predicted by the best fitting model. SFU = subjective future uncertainty; BIC*i* = integrated Bayesian information criterion. See the online article for the color version of this figure.

Two of 47 participants always selected the immediate option, rendering their discounting preferences inestimable, and consequently they were excluded from analysis (*N* = 45). The proportion of choices on which participants chose the delayed option decreased with delay, indicating participants discounted delayed payoffs (Figure 6a). Fitting the baseline discount rate, *K*, separately for the two conditions (setting *m* = 0 in Equation 12, leaving *K*, *c*, and *s* as free parameters), we found that log *K* was significantly greater in the volatile condition, mean difference in log *K* = 0.37, 95% CI [0.21, 0.53], *t*(44) = 4.62, *p* < .001, *d* = 0.69, in keeping with steeper discounting.[Fig fig6]

#### Discounting Correlated With the Subjective Cost of Uncertainty

We first tested for an effect on log *K* of a subjective cost of future uncertainty in stable and volatile conditions separately. We found that log *K* in both conditions showed the expected positive relationship with the cost of future uncertainty, (SFU × *m*), Figure 6b, panel iii; stable condition: β = 0.16, *t*(43) = 2.97, *p* = .005; volatile condition: β = 0.12, *t*(43) = 4.68, *p* < .001. In a subsequent analysis, we tested whether an effect of condition (volatile vs. stable) on discounting was greater amongst participants who: (a) showed a greater subjective increase in uncertainty between conditions and (b) were more risk averse. Here, we found evidence for an hypothesized three-way interaction, (*m* × dSFU × Condition): β = 0.10, *t*(83) = 3.04, *p* = .003, indicating that a *change* in discounting between conditions was sensitive to an added cost of future uncertainty. Main effects of *condition* (β = 0.41, *t*(83) = 4.19, *p* < .001) and risk aversion (*m*), β = 0.39, *t*(83) = 2.27, *p* = .026, were also significant. Additional terms included as covariates were not significant, [dSFU]: β = 0.03, *t*(83) = 0.32, *p* = .752; [dSFU × Condition]: β = −0.02, *t*(83) = −0.62, *p* = .539; [*m* × Condition]: β = −0.10, *t*(83) = −1.10, *p* = .275. In summary, as predicted by a volatility discounting model, both baseline discounting and a volatility-dependent shift in discounting were commensurate with participants’ subjective sensitivity to future uncertainty.

#### Volatility Discounting Correlated With Baseline Discounting

We went on to fit a volatility discounting model (Equation 12) directly to the delay discounting data, fitting both conditions with the same set of parameters. As shown in Figure 6c, this model outperformed an alternative based on concave utility. Consistent with Experiment 1, within this model baseline discounting (log *K*) was significantly correlated with volatility discounting (log *m*; Spearman ρ = 0.31, *p* = .037). That is, participants who showed greater discounting in the absence of extraneous volatility also increased their discounting more in response to volatility.

Finally, we fitted a volatility discounting model by carrying participants’ risk aversion parameters forward to fit their ITCs. Here, each participant’s estimate of *m* was carried over from their risk choices, which were fitted separately. This model, which leverages information about participant-specific risk aversion, provided a better fit to the data than a null model with *m* = 0 (higher model evidence in 41/45 participants; φ > 0.999). The fit of this model to participants’ ITCs is shown in Figure 6a. Here, an effect of condition predicted by the model derives from participants’ idiosyncratic degrees of risk aversion, measured separately.

#### Learning Rate Increased With Volatility

Rescorla–Wagner learning rates were substantially higher in the volatile condition, paired *t*(46) = 17.5, two-tailed *p* < .0001, mean α-volatile = 0.68; *SD* 0.11, and close to zero in the stable condition (mean α-stable = 0.03; *SD* 0.06). The mean learning rate in the volatile condition was close to the optimal learning rate of 0.75. We found neither a significant correlation between learning rate and log *K* (stable condition: *r* = 0.14, *p* = 0.346; volatile condition: *r* = 0.006, *p* = .966; supplemental Figure S2) nor between change in learning rate and change in log *K* (*r* = −0.14, *p* = .376).

#### Reduced Hippocampal–Prefrontal Coupling Under Volatile Reward Dynamics

We first sought to replicate previously published observations by investigating neural representation of subjective value for a delayed choice option, corresponding to the time of presentation of this delayed option. Participants who did not discount delayed reward (*N* = 8) were excluded from this analysis. Consistent with prior results (Owens et al., 2017; Wesley & Bickel, 2014), we found clusters in middle temporal gyrus (left −48 −46 7, *t* = 6.58) and dorsolateral prefrontal cortex (right 39 8 40 and 54 26 25, *t* = 6.57, extending into right anterior insula) that survived whole brain correction at family-wise error (FWE) *p* < .05.

We hypothesized that higher volatility should lead to a decrease in the reliability of simulated future scenarios. Accordingly, we predicted that medial-temporal lobe (MTL) and related regions (which support imagining future scenarios), would participate less in the evaluation of more volatile delayed rewards. In fMRI data, this predicts: (a) a decreased correlation between MTL activity and discounted value in the volatile condition compared to the stable condition and (b) an associated modulation in functional coupling between MTL and prefrontal regions tracking discounted value.

To evaluate the first of these two hypotheses, we tested for regions whose activity correlated more strongly with discounted value in the stable compared to the volatile condition. This contrast revealed activation in left amygdala (FWE corrected within a bilateral amygdala mask, −30 2 − 23, *t* = 3.65, *p* = .029; supplemental Figure S3b); however, this did not survive small volume correction for a larger a priori region of interest encompassing bilateral HC and amygdala (*p* = .11 corrected). The reverse contrast yielded no significant suprathreshold clusters at *p* < .001.

To test the second hypothesis, we performed a psychophysiological interaction (PPI) analysis with a seed in the HC, with Condition ([stable–volatile]) as a modulating variable. Although we did not observe hippocampal activation in our primary contrast of interest, we selected a hippocampal seed region on an a priori basis, since HC is known to provide for a cognitive map (Addis et al., 2007; Hassabis et al., 2007; Peters & Büchel, 2010; see supplemental material). In order to test which regions showing increased HC-coupling in the stable condition also correlated with discounted value, we used the discounted value contrast (without an effect of condition) as a mask for this PPI. For left HC-coupling, we found a significant peak in left dorsolateral prefrontal cortex (left dlPFC −39 26 25, *t* = 5.22, *p* = .025, FWE corrected for the volume of the discounted value mask), which both tracked discounted value and showed decreased coupling with HC in the volatile condition (supplemental Figure S8). The equivalent analysis for right HC-coupling yielded no significant activations. There were no significant clusters at *p* < .001 uncorrected whose connectivity with HC was greater in the volatile condition.

### Summary

Here, we illustrate effects of volatility on discounting at a time-scale commensurate with that used in conventional discounting questionnaires, across delays of up to 4 months. Our findings support a conclusion that delay discounting incorporates uncertainty discounting. We find a decrease in functional coupling under volatile conditions between MTL (HC) and a region of left dorsolateral prefrontal cortex (dlPFC), which tracked discounted value. This finding suggests that volatility-dependent increases in discounting are associated with reduced engagement of MTL structures known to participate in prospective forecasts.

## Experiment 3

Experiment 3 tested for replicability of effects in Experiments 1 and 2 in a larger, online sample. A replication test was motivated by findings that estimated correlation coefficients are unstable in smaller sample sizes (Schönbrodt & Perugini, 2013). The design followed that of Experiment 1.

### Method

#### Ethics Statement

All participants gave full informed consent before taking part in the study. The study procedures received approval from the UCL Research Ethics Committee (20399/001) and were carried out in accordance with these guidelines.

#### Data and Code Availability

Behavioral data supporting the findings of this study are publicly available online in a third-party repository: https://doi.org/10.5061/dryad.47d7wm3k2 (G. Story, 2023). Computer codes that support the findings of this study are available from the corresponding author upon reasonable request.

#### Sample Size and Power Calculation

Budget constraints limited our sample size to approximately 100 participants. Statistical simulations indicate that this sample size allows the Pearson correlation coefficient *r* to be estimated within an interval of ±0.15 with 80% confidence, for a true correlation of *r* = 0.3 (approximately the size of correlation observed between log *K* and risk aversion in Experiment 2; Schönbrodt & Perugini, 2013). Participants were recruited from Prolific.co, an online subject database.

#### Baseline Delay Discounting

Prior to starting the task, participants (*N* = 101, mean age 28.9 years, *SD* 9.7 years; 52 female) made a series of binary choices between a monetary reward of magnitude £23, £23.50, £25, or £26.50, delayed by 3, 7, 12, or 18 weeks, respectively, and a smaller quantity of money available immediately. Choices were selected according to an adaptive procedure (see supplemental material). Participants were informed that we would select one choice from every twenty participants to be paid for real as a bonus in Prolific.

#### Learning Price Dynamics

Market prices evolved according to a Gaussian random walk, upper bounded at £40 and lower bounded at £0. For one of the three products (“no volatility”), the market price was held constant at £20.00; the market price of the other two products (“low volatility” and “high volatility”) evolved with volatility σ = 1.5 and σ = 3.5, respectively. All prices were subject to Gaussian emission noise, with standard deviation η = 3. Price profiles were selected so that the market price on the final trial was equal to the long-run mean of £20.00.

#### ITC Procedure

After observing the price dynamics of each product, participants were asked to predict each product’s future price and report a subjective confidence interval, as described for Experiment 2. Eight out of 101 participants did not adjust the subjective confidence interval from its starting value in at least one of the three conditions, suggesting inattention to the task; these participants were therefore excluded. Participants subsequently chose when to sell the product, either immediately for a guaranteed price, or for the market price after a stated delay (1, 4, 9, or 13 weeks). As for Experiment 1, one choice was selected to be realized and was paid out as a bonus at the end of the experiment. An adaptive procedure was used to estimate indifference points at each delay by adjusting the immediate price (see online supplemental material). Statistical analyses followed those previously described for Experiments 1 and 2. By distinction from Experiments 1 and 2, here future sales were for the *market price*, without emission noise; we therefore set η^2^ to zero when fitting models.

### Results

#### Nonveridical Estimates of Subjective Future Uncertainty

**Figure 7 fig7:**
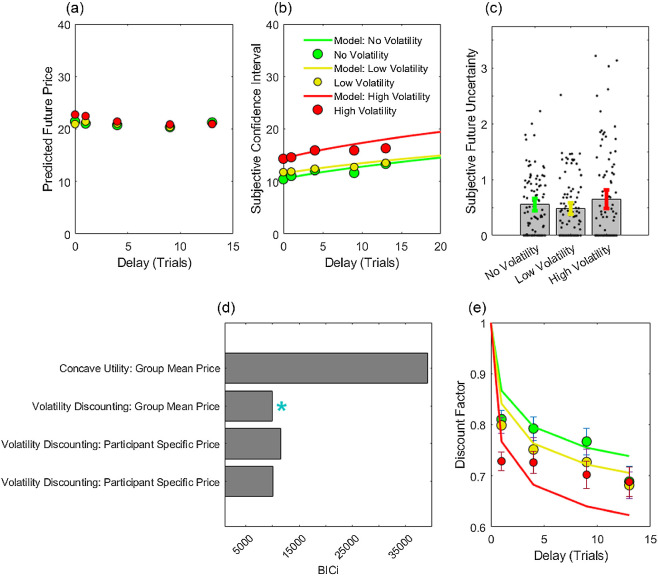
Subjective Future Uncertainty and Delay Discounting in Experiment 3 *Note*. (a) Mean “best guesses” about future prices. Participants accurately predicted that expected prices are constant across delay. (b) Mean width of subjective confidence intervals on future prices. Solid lines show the fit of a random walk model. (c) Subjective future uncertainty (SFU), that is, subjective volatility $\hat{\sigma }$ derived from growth in confidence intervals across delay. Filled gray bars show group means, solid green, yellow and red bars 95% CIs; black dots show individual parameter estimates. Participants overestimated future uncertainty in the no volatility and low volatility conditions and underestimated future uncertainty in the high volatility condition. (d) Model comparison; blue asterisk indicates the best fitting volatility discounting model. (e) Discount curves in each condition, fitted with a volatility discounting model. The model overestimated discounting in the high volatility condition, commensurate with participants’ underestimating future uncertainty in this condition. BIC*i* = integrated Bayesian information criterion. See the online article for the color version of this figure.

As shown in Figure 7a, future price predictions largely reflected a veridical pattern of no net growth or decay. Subjective confidence intervals about future prices increased as a function of delay (Figure 7b). However, unlike in Experiment 2, group average confidence intervals did not recapitulate the statistics of the true generative process. Instead, participants overestimated volatility in the no volatility condition (σ^2^ = 0, mean ${\hat{\sigma }}^{2}=0.55$) and underestimated volatility in the low volatility (σ^2^ = 1.5, mean ${\hat{\sigma }}^{2}=0.48)$ and high volatility (σ^2^ = 3.5, mean ${\hat{\sigma }}^{2}=0.65)$ conditions. There was no significant difference between subjective future uncertainty ($SFU\overset{\text{def}}{=}{\hat{\sigma }}^{2}$) in high volatility and no volatility conditions, paired *t*(92) = 1.16, two-tailed *p* = .24 (Figure 7c). However, the intercept term, corresponding to an estimate of time-independent uncertainty, was significantly higher in the high volatility condition, paired *t*(92) = 4.73, two-tailed *p* < .0001. This pattern suggests that, in this online experiment, participants either did not fully differentiate price dynamics in the three conditions, or did not fully attend to delay when providing subjective confidence intervals.[Fig fig7]

#### Delay Discounting Increased With Volatility

We fitted a volatility discounting model to participants’ choices. A model in which reward magnitude was given by mean future price predictions across participants outperformed a version in which magnitude was estimated from participants’ individual future price predictions (Figure 7d; ΔBIC*i* = 1,544, higher model evidence in 81/93 participants; φ > 0.999). This model also outperformed a null model in which *m* = 0 (ΔBIC*i* = 317; higher model evidence in 48/93 participants; φ = 0.999), supporting an effect of volatility to increase delay discounting. Finally the volatility discounting model outperformed an alternative based on concave utility (ΔBIC*i* = 2000; higher model evidence in 93/93 participants; φ > 0.999). Discount curves for the three products are shown in Figure 7e, plotted using mean future price predictions as estimates of future reward magnitude. As predicted, discounting increased in proportion to volatility, linear mixed effects on log *K* fitted to each condition separately: β_condition_ = 0.04, *t*(277) = 4.13, *p* < .001.

#### Baseline Discount Rate Correlated With In-Task Volatility Discounting

**Figure 8 fig8:**
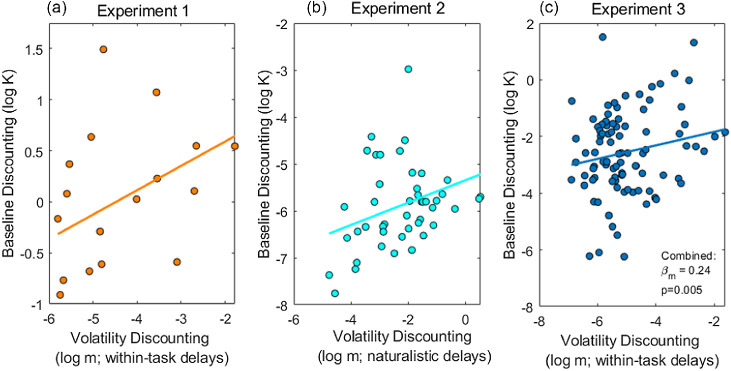
Baseline Discounting Versus In-Task Volatility Discounting *Note*. (a) For Experiment 1, volatility discounting (log *m*), that is, sensitivity of discounting to volatility within-task, correlates with baseline discounting for nonrisky rewards measured outside of the task (*N* = 17; three participants excluded due to inestimable baseline discounting data). (b) In Experiment 2 (*N* = 45), where in-task discounting was measured over naturalistic timescales, volatility discounting (log *m*, estimated from discounting choices) correlates with baseline discounting (log *K*_0_, discounting in stable condition). (c) In Experiment 3 (*N* = 93), in-task volatility discounting (log *m*) correlates with discounting for nonrisky rewards measured outside of the task. Solid lines show fits of a linear mixed-effects regression with experiment as a random grouping variable, fixed slope β_*m*_ = 0.24, *t*(153) = 2.84, *p* = .005. See the online article for the color version of this figure.

Volatility discounting within the task, governed by parameter *m*, showed a significant positive correlation with baseline discounting, measured outside of the task (log *m* vs. log *K*: Pearson *r* = 0.22, *p* = .035). As shown in Figure 8, this finding was consistent across all three experimental tasks. Pooling data across the three experiments revealed a highly significant positive relationship between log *m* and log *K*, linear mixed-effects regression with random slope and intercept parameters, grouped by experiment: fixed slope β_*m*_ = 0.24, *t*(153) = 2.84, *p* = .005.[Fig fig8]

#### Learning Rate Increased With Volatility

Learning rates increased overall with increasing volatility, linear mixed effects on α: β_condition_ = 0.32, *t*(277) = 6.16, *p* < .001. However, the pattern was nonmonotonic, suggesting that participants did not fully distinguish between no volatility and low volatility conditions (no volatility: mean α = 0.16, *SD* = 0.43, low volatility, mean α = 0.05, *SD* = 0.60, high volatility mean α = 0.77, *SD* = 0.88). We speculate that this was due to inattention to the task. It is also possible that increased emission noise in this experiment (relative to Experiment 2) obscured participants’ perceptions of volatility. As previously, in-task discounting showed no significant correlation with learning rate across participants in any of three conditions (no volatility: *r* = −0.02, *p* = .815; low volatility: *r* = −0.08, *p* = .422; high volatility: *r* = −0.19, *p* = .051; supplemental Figure S5).

### Summary

In summary, Experiment 3 reproduced findings that in-task delay discounting increased with increasing volatility, to an extent that correlated with baseline discounting. Notably, here participants’ subjective estimates of future uncertainty and learning rate during the task did not reflect the true reward statistics, perhaps suggesting lower attention to the task during this online experiment than was achieved by participants in the previous laboratory experiments.

## General Discussion

A central idea in behavioral economics considers impatience as arising, at least partly, from risk implicit in a delay. Existing approaches have emphasized that delayed rewards are less probable than immediate ones, rather than that the value of delayed reward is less precisely known. By contrast, here, we advance a model in which random changes in reward value accumulate over time. If risky rewards are discounted according to their variance, this model yields impatience, to an extent that is proportional to volatility (changeability) of reward value. This model has affinities with concepts found in finance, such as the *price of risk*, the excess rate of return on an investment demanded to compensate for an increase in volatility, expressed per unit of volatility (Sharpe, 1964). Consistent with volatility discounting, we find that people discount delayed reward more steeply in volatile environments, an effect that also pertains over naturalistic delays of up to 4 months.

In the experiments presented here, objective outcomes (prices) are uncertain, whereas in conventional discounting tasks the objective outcomes are certain (e.g., $10 or $20 tomorrow), but *subjective* values are uncertain. It may be argued that manipulating volatility experimentally in this manner simply adds extraneous risk to a baseline delay discounting process, rather than revealing that subjective values incorporate estimates of volatility. However, three findings mitigate this concern. First, in the stable condition of Experiment 2, where there is no extraneous time-dependent risk, delay discounting nevertheless correlated significantly with a subjective cost of future uncertainty. Second, participants who showed steeper discounting in the stable condition also increased discounting more in response to volatility. Third, within-task volatility discounting in Experiments 1 and 3 correlated with delay discounting of nominally riskless rewards *outside* of the task. Taken together, these findings support a conclusion that impatience for reward is partly determined by an aversion to future uncertainty. Nevertheless, the experiments presented here manipulate volatility explicitly, so participants may have been primed to attend to this. Future work might examine effects on discounting of implicit manipulations of volatility.

We do not suggest that volatility is the sole factor underlying delay discounting. Rather, normative factors independent of risk, such as an opportunity cost associated with delay, ought to affect discounting (Niv et al., 2007; Rachlin, 2006), perhaps accounting for observations that probability and delay discounting are subject to distinct influences (Cox et al., 2020; Estle et al., 2006; Lee et al., 2020; Luhmann et al., 2008; Peters & Büchel, 2009; Weber & Huettel, 2008). Furthermore, while volatility pertains to uncertainty over the magnitude of future reward, in conventional discounting tasks both the probability of receipt (e.g., Sozou, 1998) and the timing of the receipt may also be uncertain (Dasgupta & Maskin, 2005).

### Sampling Future Reward in Prospective Memory

Gabaix and Laibson (2017) propose that, owing to time-dependent uncertainty, mental simulations of future events are less precise than more immediate ones. The formal implications for discounting are similar to those considered here. A key hypothesis is that cognitive resources ought to be devoted to simulating the future only where doing so is worth the reward gained and is effective in reducing future uncertainty (Gershman & Bhui, 2020). Our imaging findings are consistent with this idea. In Experiment 2, we investigated volatility-dependent increases in discounting in terms of relative engagement of MTL structures known to participate in prospective forecasts, in particular, the HC (Addis et al., 2007; Hassabis et al., 2007; Johnson & Redish, 2007; Peters & Büchel, 2010; Schacter et al., 2008; Tsao et al., 2018). We found a decrease in functional coupling under volatile conditions between HC and a region of left dorsolateral prefrontal cortex (dlPFC), which tracked discounted value.

Anatomically, dlPFC is reciprocally connected to HC via the parahippocampal gyrus, subiculum, and presubiculum (Goldman-Rakic et al., 1984) and is a region implicated in the exercise of cognitive control (Hare et al., 2009; Hare et al., 2009; Hecht et al., 2013; MacDonald et al., 2000; Rudorf & Hare, 2014). An interpretation is therefore that dlPFC maintains an online representation of delayed reward, by retrieving contextual information from MTL, and this coupling is diminished under unpredictable reward dynamics. We tentatively suggest that this effect reflects a cost-benefit trade-off whereby imagining the future is more effortful and is therefore downweighted. This finding is in keeping with a previous report that in a reinforcement-learning task, dlPFC was more strongly activated under predictable than under unpredictable state transition rules (Tanaka et al., 2006). Notably however our primary contrast of interest did not reveal hippocampal activation, placing our connectivity analysis on a relatively weak evidential footing.

### Effects of Nonlinear Utility and Internal State

In economic models, risk aversion is often accounted for by postulating a concave utility function over reward magnitude, such that rewards of increasing magnitude are associated with decreasing marginal benefits (Bell, 1995; Kahneman & Tversky, 1979; Pine et al., 2009). We found that a volatility discounting model outperformed a model based on a concave power law utility function. However, effects of nonlinear utility cannot be fully delineated from the model presented here, since the two effects both converge on a prediction that discounting depends on the variance of future reward. Indeed, it is likely that there exists some alternative form of utility function that would approximate hyperbolic discounting of variance.

In the experiments presented here, we manipulate volatility in external reward magnitude. However, the same model might also be applied to changes in the subjective utility of reward deriving from changes in internal state. For example, a person asked to specify what they would like to eat for dinner a week in advance might be unsure of whether their preferences will be the same in a week’s time. Effects of internal state changes on both risk attitude (Kacelnik, 1997; Smallwood, 1996) and delay discounting (Giordano et al., 2002; Kirk & Logue, 1997; Mitchell, 2004) are well documented and examining the interactions of such effects with those of future uncertainty suggests an important direction for future research.

### Learning Rate and Discount Rate as Separable Markers of Impulsivity

As predicted by optimality, volatility-dependent increases in temporal discounting seen here were also associated with an increase in learning rate (Behrens et al., 2007; Iigaya, 2016; Nassar et al., 2010, 2012). In reinforcement-learning models, the value of an action is estimated by updating a recency-weighted average of rewards which followed that action in the past (Mathys et al., 2011; Wilson et al., 2010), where a high learning rate entails steeper discounting of past rewards and faster value updates (Rescorla & Wagner, 1972; Sutton & Barto, 1998). A high setting of either rate can generate impulsive behavior: A high learning rate leads to behavior driven by recent successes or failures, rather than the long-run context, while a high discount rate produces behavior driven by its immediate consequences, thereby neglecting delayed benefits. We show that volatility engenders an increase in both parameters, reflecting the fact that where reward contingencies change frequently, neither past rewards nor promised future rewards are fully informative of current action value (Daw et al., 2005, 2006; Dolan & Dayan, 2013).

That we did not find a correlation between learning rate and discount rate across participants suggests that future discounting engages processes different to those involved in learning. In particular, we find that while learning rate is sensitive to volatility, discounting is sensitive to an *interaction* between volatility and risk aversion. Within this interaction, risk aversion appears to contribute the greater source of between-participant variability. An additional consideration is that the form of weighted averaging over past rewards predicted by Rescorla–Wagner learning does not correspond directly to the model used here for future discounting. Further work might examine whether integration of past and future rewards might be fitted with the same model.

Finally, we note that increasing time-independent uncertainty ought to *decrease* both learning rate (Mathys et al., 2011; Wilson et al., 2010) and delay discounting (by Equation 8 and supplemental Equation S12). This accords with findings that adding risk to both immediate and delayed rewards reduces a bias toward immediate reward (Anderhub et al., 2001; Andreoni & Sprenger, 2012b; Keren & Roelofsma, 1995; Stevenson, 1992; though see Ahlbrecht & Weber, 1997) and that increasing emission noise reduces learning rate (Diederen & Schultz, 2015). In this regard, an interesting direction for future research would be to examine a tendency to misperceive emission noise as volatility, which may underpin maladaptive impulsivity. For example, in learning tasks, people with high trait anxiety show smaller adjustments in learning rate in response to changing volatility (Browning et al., 2015), and increased reliance on “lose-shift” strategies (Huang et al., 2017). These findings suggest that anxious individuals may misperceive chance fluctuations as underlying environmental changes, particularly following losses. Further research might explore whether this effect correlates with the increased delay discounting reported amongst anxious individuals (Xia et al., 2017).

### Conclusions

We conclude that individual differences in delay discounting in part reflect differential discounting of uncertainty, whereby more impatient people are more sensitive to future risk. The present study contributes to a growing body of work illustrating how discounting can be derived from beliefs about the statistics of future reward across time (e.g., Patak & Reynolds, 2007; Reynolds et al., 2007; Stevens et al., 2005; Takahashi et al., 2007). We propose that measuring such beliefs directly offers to enrich our understanding and prediction of impulsive behavior. This idea can also reconcile state- and trait-level influences on discounting in that, over long timescales, prevailing environmental conditions ought to be reflected in trait-based differences in discounting, which are subject to state-based effects when conditions change (Griskevicius et al., 2011; Haynes et al., 2021; Koffarnus et al., 2013; Odum, 2011; Peviani et al., 2019). However, more integrative studies are required to examine how the various documented influences on discounting interact to shape impulsive behavior “in the wild.”

## Supplementary Material

10.1037/dec0000219.supp
