# Supplementary material for: Discounting Future Reward in an Uncertain World
Source: Decision (Wash D C ). 2023 Jun 29;11(2):255–82. doi: 10.1037/dec0000219 (PMC11949085; doi:10.1037/dec0000219)
Supplement: Supplementary file 1 [file DEC-2022-0233_Supplemental_materials_dec0000219.docx]

Discounting Future Reward in an Uncertain World

Supporting Information Online

G. W. Story^1^, Z. Kurth-Nelson^1,2^, M. Moutoussis^1,3^, K. Iigaya^1,4,5^, G.-J. Will^3,6^, T. U. Hauser^1,3^,B. Blain^8,1^, I. Vlaev^7^, R. Dolan^1,3^

1 Max Planck UCL Centre for Computational Psychiatry and Ageing, UCL, UK

2 DeepMind, UK

3 Wellcome Centre for Human Neuroimaging, UCL, UK

4 Gatsby Computational Neuroscience Unit, UCL, UK

5 Division of Humanities and Social Sciences, California Institute of Technology, USA

6 Institute of Psychology, Leiden University, Netherlands

7 Warwick Business School, University of Warwick, UK

8 Department of Experimental Psychology, UCL, UK

**Bayesian Interpretation of Volatility Discounting Model**

To derive volatility discounting from Bayesian principles, we treat stated future rewards as samples from an underlying generative distribution (see also Gabaix & Laibson, 2017; Gershman & Bhui, 2020). We propose that a decision-maker computes an average of the magnitude of promised future rewards. A key aspect of our model is that uncertainty about each reward’s magnitude grows with delay. As a result, distant rewards are down-weighted when calculating this average.

Formally, we posit that at a decision-maker computes a posterior distribution over *average reward* per time step,$\mu$, given by:

$$p\left( \mu| \boldsymbol{x} \right)\propto p\left( \mu\right)p\left( \boldsymbol{x} | \mu\right)$$

(S1)

Where $\boldsymbol{x}$ is a vector of stated future reward magnitudes:

$$\boldsymbol{x}={\{x}_{0},x_{1},x_{2}\ldots x_{T}\}$$

(S2)

We first consider a simple case in which the prior, $p\left( \mu\right),$is uniform, i.e. average future reward is estimated from the prospective data alone. The likelihood function is given by:

$$p\left( \boldsymbol{x} | \mu\right)=\prod_{t=0}^{T} p(x_{t}|\mu)$$

(S3)

As described in the Main Text, we consider a generative model in which reward magnitude, $x$, is a Gaussian random variable. Specifically, stated reward magnitude in each time period, $x_{t}$, is treated as a single sample from a Gaussian distribution, the variance of which depends on delay, such that (by Equation 5 in the Main Text):

$$p\left( x_{t} | \mu\right)\mathbb{\sim N(}x; \mu,t\sigma^{2} +\vartheta^{2})$$

(S4)

We assume the decision-maker knows the noise parameters, $\sigma^{2}$ and $\vartheta^{2}$. By Bayes’ rule for Gaussians the posterior mean is given by an average of observations, weighted according to their precision:

$$\hat{\mu}≝\hat{V}\left( \boldsymbol{x} \right)=\sum_{t=0}^{T} {\frac{\pi_{t}}{\sum_{t} \pi_{t}}x}_{t}$$

(S5)

Where precision is given by:

$$\pi_{t}=\frac{1}{t\sigma^{2} +\vartheta^{2}}$$

(S6)

In other words, when computing average reward, the decision maker combines promised future rewards, down-weighting each according to its uncertainty. Distant rewards are treated as more uncertain (noisier) estimates, and therefore receive less weight.

The values of an immediate reward of stated magnitude, $x_{0}$, available at time $t=0$, and a delayed reward of stated magnitude, $x_{t}$ available at time $t>0$, are given by:

$$\hat{V}\left( x_{0} \right)=x_{0}\frac{\pi_{0}}{\sum_{t} \pi_{t}}$$

$$\hat{V}\left( x_{t} \right)= x_{t}\frac{\pi_{t}}{\sum_{t} \pi_{t}}$$

(S7)

Note that rewards in remaining periods have expected magnitude zero, and therefore do not contribute to the posterior mean. At indifference therefore:

$$x\frac{\pi_{0}}{\sum_{t} \pi_{t}}=x_{t}\frac{\pi_{t}}{\sum_{t} \pi_{t}}$$

(S8)

The total precision terms cancel to give:

$$\frac{x_{0}}{x_{t}}=\frac{\pi_{t}}{\pi_{0}}$$

(S9)

For a case where immediate and delayed rewards have equal emission noise:

$$\frac{x_{0}}{x_{t}}=\frac{\vartheta^{2}}{{(\vartheta}^{2}+t\sigma^{2})}$$

(S10)

Dividing the right hand side by $\vartheta^{2}$ gives:

$$\frac{x_{0}}{x_{t}}=\frac{1}{1+Kt}$$

(S11)

Where:

$$K=\frac{\sigma^{2}}{\vartheta^{2}}$$

(S12)

That is, hyperbolic discounting, with rate proportional to time-dependent uncertainty (volatility), and inversely proportional to time-independent uncertainty (emission noise). We accordingly term this model *volatility discounting*. This arrangement accords with findings that adding risk to both immediate and delayed rewards reduces a bias towards immediate reward (Andreoni & Sprenger, 2012b; Keren & Roelofsma, 1995; Stevenson, 1992; Anderhub et al., 2001; though see Ahlbrecht & Weber, 1997).

For a case where immediate reward is nominally certain (by Equation 7 in the Main Text):

$$\frac{x_{0}}{x_{t}}=\frac{\theta^{2}}{{\theta^{2}+\eta}^{2}+t\sigma^{2}}$$

(S13)

Dividing by $\theta^{2}$ and substituting $m=1/\theta^{2}$ gives:

$$\frac{x_{0}}{x_{t}}=\frac{1}{{1+m(\eta}^{2}+{t\sigma}^{2})}$$

(S14)

Thus, the same arrangement yields hyperbolic discounting of rewards according to their variance, with rate *m*.

**Relationship with Previous Models**

Recently published accounts derive hyperbolic discounting from a model similar to that outlined above (Gabaix & Laibson, 2017; Gershman & Bhui, 2020). In the aforementioned models time-dependent uncertainty is seen as arising from noise in internal simulations of future reward, rather than from volatility in the true generative process. The formal implications of external and internal volatility are similar. However, our model differs in its treatment of precision.

Gabaix and Laibson (2017), primarily illustrate a situation in which decision-makers take a precision-weighted average of a single future reward estimate and a prior mean, $\rho$. Shown in the current notation:

$$\hat{V}\left( r_{t} \right)=\omega_{\rho}\rho+{\omega_{t}r}_{t}$$

(S15)

Where:

$$\omega_{\rho}=\frac{\pi_{\rho}}{\pi_{\rho}+\pi_{t}}$$

$$\omega_{t}=\frac{\pi_{t}}{\pi_{\rho}+\pi_{t}}$$

(S16)

And the prior is Gaussian, such that:

$$p\left( \mu\right)\mathbb{\sim N(}\rho,\varphi^{2})$$

$\pi_{\rho}=1/\varphi^{2}$

(S17)

A corollary of this arrangement is that the value of future reward returns to the prior mean at long delays, as $\pi_{t}$ tends to zero. Gabaix and Laibson (2017) show that this leads to a discount rate given by the ratio of prior precision and data precision. For example, focusing on the case where the prior mean ($\rho)$ is zero:

$$\hat{V}\left( r_{t} \right)=r_{t}\frac{\pi_{t}}{\pi_{\rho}+\pi_{t}}$$

(S18)

Which gives hyperbolic discounting where $K=\pi_{\rho}/\pi_{t}$.

By contrast, here we focus on a choice between immediate and future reward, where immediate reward also entails some uncertainty. Where prior means and precisions are equal for both rewards, their effect cancels out during intertemporal choice. By Bayes’ rule for Gaussians the posterior mean is given by a weighted average of the prior and sampled data:

$$\hat{\mu}≝\hat{V}\left( \boldsymbol{r} \right)=\omega_{\rho}\rho+\sum_{t=0}^{T} {\omega_{t}r}_{t}$$

(S19)

Where:

$$\omega_{\rho}=\frac{\pi_{\rho}}{\pi_{\rho}+\sum_{t} \pi_{t}}$$

$$\omega_{t}=\frac{\pi_{t}}{\pi_{\rho}+\sum_{t} \pi_{t}}$$

(S20)

Using the binary choice example above, at indifference:

$$\rho\frac{\pi_{\rho}}{\pi_{\rho}+\sum_{t} \pi_{t}}+ r_{0}\frac{\pi_{0}}{\pi_{\rho}+\sum_{t} \pi_{t}}=\rho\frac{\pi_{\rho}}{\pi_{\rho}+\sum_{t} \pi_{t}}+ r_{t}\frac{\pi_{t}}{\pi_{\rho}+\sum_{t} \pi_{t}}$$

(S21)

Taking the ratio, it can easily be shown that the prior means and precision terms cancel, to give, as previously:

$$\frac{r_{0}}{r_{t}}=\frac{\pi_{t}}{\pi_{0}}$$

(S22)

Thus, in our framework, while the prior mean influences the discounted value of a reward considered in isolation, the relative value of two rewards with equal prior means is influenced only by their relative precisions.

**Normalization of Values**

In the notation above, values are normalized by the total precision, to provide for an estimate of *average* reward. We denote such normalized values as $\hat{V}\left( \boldsymbol{r} \right)$. In the Main Text we use a notation without normalization, such that:

$$V\left( \boldsymbol{r} \right)=\hat{V}\left( \boldsymbol{r} \right)\sum_{t} \pi_{t}=\sum_{t=0}^{T} {\pi_{t}r}_{t}$$

(S23)

Normalisation leaves the ratio of immediate and delayed reward, and hence the form of discount function, unaffected: reward magnitudes are rescaled by a factor that depends on the total precision, $\sum_{t} \pi_{t}.$

**Supporting Information: Experiment 1**

**Supporting Methods**

***Trial Structure of Learning Phase***

For a ‘No Volatility’ product the market price was held constant. The market price of the other two products (‘Low Volatility’ and ‘High Volatility’) underwent random changes across time. Price trajectories for these two products were simulated by implementing a time-dependent probability that the market price would change to a new value, selected from a uniform distribution between specified bounds. For a ‘Low Volatility’ product, changes in the market price were small, while for a ‘High Volatility’ product, changes were more extreme.

Within each block, participants performed three phases of observation and prediction: the first consisted of 70 observation trials followed by 70 prediction trials, while the subsequent two phases each consisted of 45 observation trials and 5 prediction trials. After each phase the price evolution was paused whilst participants made a set of intertemporal choices. Learning rates were fitted based on the first 70 prediction trials; subsequent prediction phases were included to ensure that participants attended to prices before making intertemporal choices.

***Learning Behaviour***

We estimated learning rates for the two items separately by fitting a Rescorla-Wagner learning model (Rescorla & Rescorla, 1967) to their price predictions, of the form:

$$\hat{R}_{t} = \hat{R}_{t-1} + \alpha(r_{t}- \hat{R}_{t-1})$$

(S24)

$\hat{R}_{t}$, the estimated market price at week *t*, is given by the previous estimate plus an update proportional to the prediction error between the previous estimate and the observed price this week, $r_{t}$. The learning rate, $\alpha$, governs the extent to which this prediction error influences subsequent estimates. The likelihood of a participants’ price estimate on each trial was given by a truncated Gaussian distribution, centred on $\hat{R}_{t}$, where the precision of this distribution was a free parameter. Learning rates were estimated using data from the first block of 70 prediction trials.

***Volatility Discounting Model and Concave Utility Model***

We fitted participants’ in-task intertemporal choices with a Volatility Discounting model. The model assumed two independent contributions to discounting: a baseline component due to effects other than volatility, with rate $K$, and an additional component due to volatility. Here, we calculated $\sigma^{2}$ from the slope of a relationship between the objective price variance and delay.

We also tested a different class of model wherein risk preference is accounted for by concave utility over reward magnitude. This model assumes that participants estimate the distribution of future reward, by chunking reward into four bins (corresponding to quartiles of the permitted range) and approximating reward magnitudes as the bin centers. The probability mass of each bin is calculated by integrating a Gaussian distribution, centered on a participant’s future price estimate, with variance governed by volatility at the relevant delay. The expected utility of future reward is then calculated using a concave utility function, wherein $U\left( r_{t} \right)={(r_{t})}^{z}$, where $0.1<z<1$ governs risk aversion. For example, in Experiment 2 where prices can range from £0-50, the model estimates the probability that the future price will lie in each of the intervals [0-12.25], [12.26-25], [25.01-37.50], [37.51-50]. The magnitude of each interval is approximated as the midpoint. The subjective value of the future option is then computed by passing prices through the utility function, before taking the expected value.

**Supporting Information: Experiment 2**

**Supporting** **Methods**

***Training Phase***

Participants learned how the prices of two electrical products (headphones and radios) evolved week-by-week, where a week corresponded to a trial of the experiment (Figure 4). Participants first passively observed the price of each product, displayed on a linear scale ranging from £0 to £50, as it evolved over the course of 240 trials. Over a further 240 trials they were asked to predict upcoming prices. Prices for the two products were displayed in randomly ordered mini-blocks of 60 trials in length; at the start of each block the market price was reset to £25. Price predictions followed the same procedure as in Experiment 1. For the Stable product, participants were instructed the future market price would remain constant at £25, whereas for the Volatile product the future market price would drift according to the very same process they had previously observed. In both conditions, future prices were also subject to the same degree of emission noise.

***fMRI Data Acquisition***

Owing to upgrades to the scanners during data collection, MRI data were acquired on two 3T Siemens scanners (18 participants on each scanner), using a 32-channel head-coil. 176 volumes were acquired for each of four sessions (36 trials per session), and the first five volumes discarded to compensate for T1 relaxation effects. 48 slices were acquired per volume, aligned to a line connecting anterior and posterior commissures, with a voxel size of 3 x 3 x 3 mm (TR = 3.36 s, TE = 30 ms, echo spacing 0.5ms). An additional T1 weighted MDEFT structural image was acquired for anatomical normalisation (voxel size 1 × 1 × 1 mm). Subjects viewed the task through a head-coil-mounted mirror.

***fMRI Data Analysis***

Image processing and statistical analysis was performed using SPM12 (Functional Imaging Laboratory, University College London). All volumes were spatially realigned to the first volume to remove effects of subject movement, using a 6-parameter affine transformation, and unwarped to correct for non-linear field distortions as a function of subject movement. The high-resolution structural T1 image was co-registered to the functional images, which were in turn spatially normalized to MNI space, and finally smoothed with an isotropic Gaussian kernel of 8 mm full-width at half-maximum, to allow for parametric statistical inference at the group level.

We modelled fMRI data using a general linear model (GLM) implemented in SPM12. The presentation of both immediate and delayed options separately were modelled by convolving the duration of stimulus presentation with the canonical haemodynamic response function (HRF) as a box-car regressor, separately for each session. We included a factor for the effect of Condition (coded as -1 for Volatile and 1 for Stable), as well as parametric regressors for the subjective value of each option. Discounted value of the delayed option was calculated as the expected magnitude of the delayed option (£25 for all choices) multiplied by the group mean discount factor estimated from a model with a single discount rate (K) across both Volatile and Stable conditions. Group-mean rather than participant-specific parameters were used, so that individual differences in BOLD response to discounted value loaded onto the GLM regression weights. Similarly, we used a single discount rate rather than separate rates for the two conditions, to allow differences between conditions to be captured by the regression weight on subjective value in the GLM. Additional parametric regressors of no interest coding for the six movement parameters were included in the GLM. To increase sensitivity to detect correlations with discounted value at the group level we excluded participants (N=8) who displayed no significant degree of discounting. Contrast images were generated for the effects of condition and for the parametric effects of subjective value, and entered into a second-level analysis in SPM 12, with scanner identity as a between-subject covariate (coded -1 and 1). For this analysis we examined an *a priori* region-of-interest (ROI) encompassing amygdala and hippocampus. This ROI was selected based on previous reports that episodic future thinking is associated with increased functional connectivity between hippocampus/amygdala and pre-frontal regions encoding discounted value (Peters and Buchel, 2010).

We used the same GLM for a Psycho-Physiological Interaction (PPI) analysis. To define the seed region we repeated the contrast examining for the effect of condition [Stable – Volatile] on discounted value, at a highly relaxed threshold of p<0.05 uncorrected, within a region of interest encompassing hippocampus and amygdala. In addition to the activations reported above, this revealed peaks in bilateral hippocampus (HC) (left -18 -13 -17, *t*=2.74, p=0.005 uncorrected; right 24 -10 -20, *t*=2.20, p=0.018 uncorrected), which we selected as seed regions. Time courses were extracted from this seed region, and corrected for all contrasts of interest. For each participant the existing Condition regressor (coded as -1 for Volatile, 1 for Stable), the ROI time course and the PPI (interaction) regressor were then entered into a first level model. Contrast images were generated for the PPI regressor, which were analyzed at the second level using a one-sample t-test.

***Description of Emission Noise in the Learning Phase***

During the learning phase, participants were explicitly instructed about two sources of variability in prices: an irreducible Gaussian noise (*𝜎=2)* applying equally to both items, which we described as ‘variability in online bidding’, and drift in the underlying ‘market price’. To facilitate this explanation, in a practice phase participants first observed a series of trials in which the market price was displayed as a horizontal bar on the price scale, together with a dot indicating the highest bid. This was shown first for the Stable item, where the market price was constant, to familiarize participants with ‘variability in the bidding’, before they observed a drifting market price for the Volatile item, with the same level of variability in bidding. The horizontal bar denoting the market price was not shown during the experiment itself. Participants were told that their task during the experiment would be to predict the price on subsequent weeks and that the best way to do so is to estimate the underlying market price.

The underlying market price of the Volatile item was not revealed to participants but they were told that the market price would ‘drift up and down over time’, and that they needed to keep track of this. Participants were also instructed that due to emission noise in prices, a given price provided imprecise information about actual market price and to make good predictions they should accumulate information over a number of recent prices.

***Fitting Baseline Risk Preferences***

We fitted participants’ risk choices with a risk discounting model of the form shown in Equation 10 (Main Text), where $t=0$, and *m* captures individual differences in *risk aversion*, with the variance of a risky prospect given by $\eta^{2}$. We also tested a standard mean-variance model (Bell, 1995; d’Acremont & Bossaerts, 2008; Kroll et al., 1984; Symmonds et al., 2011; Weber et al., 2004) of the form:

$$x=X-b\eta^{2}+c$$

(S25)

Here the slope parameter, $b$, indexes risk aversion, and $c$ is a bias term.

***Subjective Future Uncertainty***

To test the veridicality of participants’ confidence intervals, we fitted a model based on the true generative process, i.e. a Gaussian random walk, specifically given by:

$$\frac{1}{2}CI=1.65 \sqrt{\hat{\sigma}^{2}t+\hat{\eta}^{2}}$$

(S26)

Where $\hat{\sigma}^{2}$ represents a participant’s inferred subjective estimate of volatility, and $\hat{\eta}^{2}$ their estimate of a fixed emission noise; note that a 90% confidence region corresponds to an area between the 5^th^ and 95^th^ percentiles, equivalent to 1.65 standard deviations either side of the mean. We termed $\hat{\sigma}^{2}$ ‘Subjective Future Uncertainty’, (SFU).

**Supporting Information: Experiment 3**

**Supporting Methods**

***Adaptive Discounting Procedure***

To estimate baseline discounting, participants made a series of binary choices between a monetary reward of magnitude £23, £23.50, £25 or £26.50, delayed by 3, 7, 12 or 18 weeks respectively, and a smaller quantity of money available immediately. Choices were selected according to an adaptive procedure. At each iteration of the procedure, choices were offered between an immediate reward and each of the four delayed rewards, presented in a random order. On the first iteration, if a participant selected the immediate reward at a given delay, the immediate reward at that delay was decreased for the subsequent iteration to a minimum value; accordingly, if a participant selected the delayed reward, the immediate reward was increased to a maximum value. Minimum values for each of the four delays respectively were £2.10, £2.20, £2.00 and £2.30; maximum values were £23.00, £23.50, £25.00 and £26.30. The size of adjustment in immediate reward halved after each iteration, to converge on participants’ indifference points. Eight iterations were performed, creating 32 choices in total. On the first iteration, the immediate reward was set to £17.50. An equivalent procedure was used to estimate *within-task* discounting, with the exception that the delayed reward option was described as ‘Selling on the market’, rather than with a specified amount. Here immediate reward was lower bounded at £5.10 and upper bounded at £29.05 for all delays.

***Learning Price Dynamics***

Prices for the three products were displayed in randomly ordered blocks; a warning screen indicated the start of each block. For each product, 40 ‘observation trials’ were followed by 60 ‘prediction trials’.

**Supporting Figures**

**Figure S1**

*Learning Rate vs Discount Rate in Experiment 1*
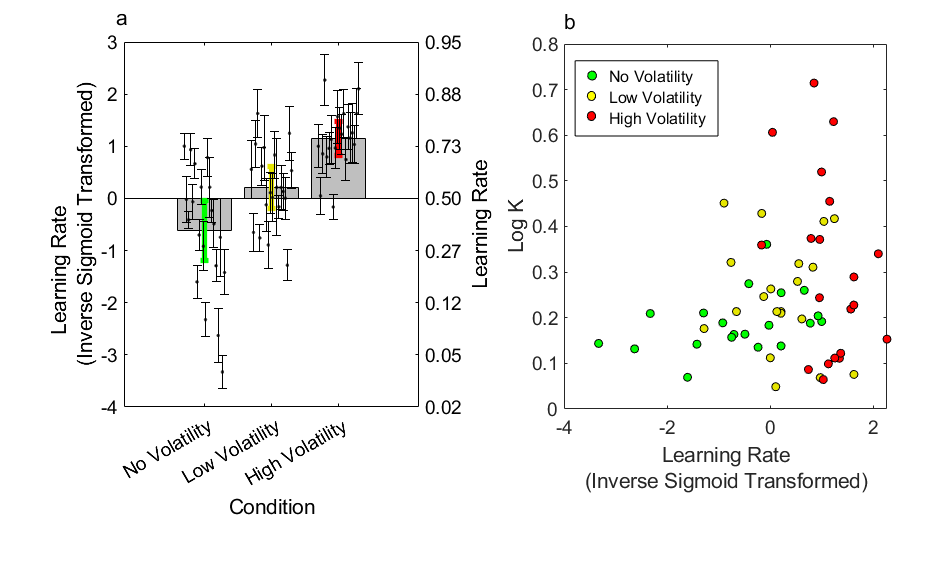


*Note.* a) Learning rate across the three conditions. Filled grey bars show group means, solid coloured bars 95% CIs; black dots show individual parameter estimates with within-subject standard errors. b) Log K plotted against learning rate across the three conditions. No between-participant correlations (within condition) reached significance at p<0.05.

**Figure S2**

*Learning Rate vs Discount Rate in Experiment 2*
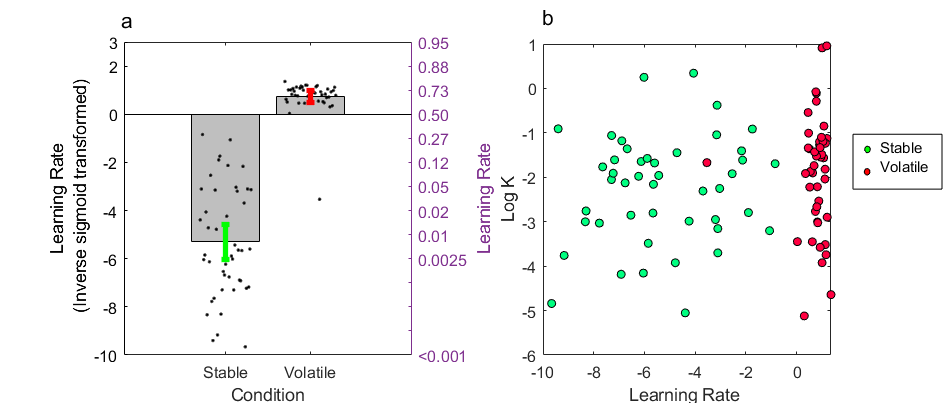


*Note.* a) Learning rate across the two conditions. Filled grey bars show group means, solid coloured bars 95% CIs; black dots show individual parameter estimates. b) Log *K* plotted against learning rate across conditions. No between-participant correlations (within condition) reached significance at p<0.05.

**Figure S3**

*Neural Responses to Discounted Value in Experiment 2*


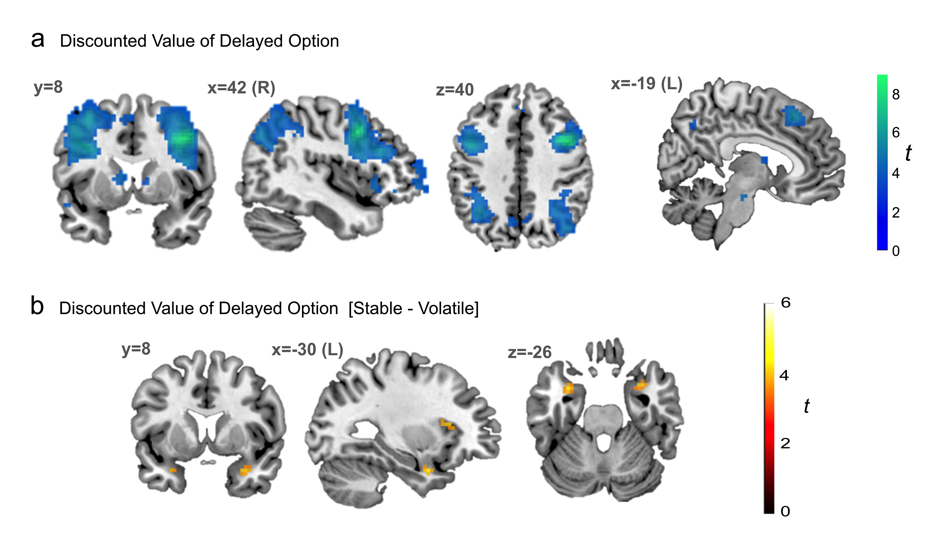


*Note.* a) BOLD correlation with discounted value of the delayed option across both conditions (in participants with positive discounting, *N*=28), thresholded at p<0.001 uncorrected for display purposes. Clusters in middle temporal gyrus (left -48 -46 7, *t*= 6.58) and dorsolateral prefrontal cortex (right 39 8 40 and 54 26 25 *t*= 6.57, extending into right anterior insula) survived whole brain correction at Family Wise Error (FWE) p<0.05. These areas align well with those previously shown in meta-analysis as responsive to the value of delayed rewards. b) Activity in bilateral medial temporal lobe, extending into entorhinal cortex (left -30 5 -26, *t*=4.35; right 33 8 -26, *t*=4.05) and left anterior insula (-33 26 10, *t*=4.31), correlated more strongly with discounted value in the Stable, relative to Volatile condition (*N*=28, displayed at p<0.001 uncorrected). A peak in left amygdala survived family wise error correction within a bilateral amygdala mask (-30 2 -23, *t*=3.65, p=0.029).

**Figure S4**

*Reduced Dorsolateral Prefrontal-left Hippocampal Coupling Under Future Uncertainty*


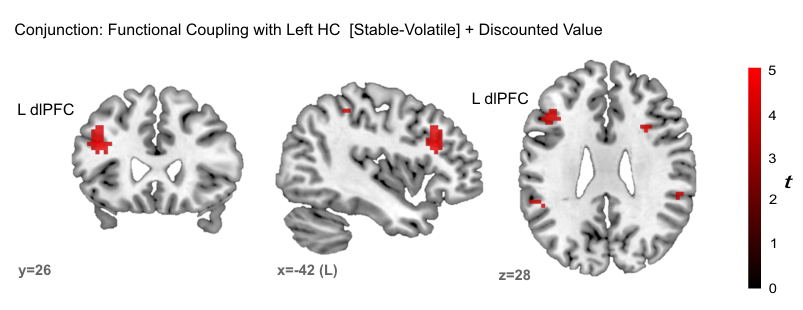


*Note.* A seed for the psychophysiological interaction (PPI) analysis was set as a functionally defined ROI in left hippocampus (HC). Results were masked to include only areas also correlated with discounted value. Functional coupling with left dorsolateral prefrontal cortex (left dlPFC -39 26 25, *t*=5.44, p=0.002 FWE corrected for volume of the mask, Bonferroni corrected for two seed regions), dorsomedial prefrontal cortex/supplementary motor area (-3 8 55, *t*=4.27, p=0.048 corrected) and parietal cortex (left -9 -64 58, *t*=5.54, p=0.002 corrected; right 45 -40 43, *t*=5.44, p=0.002 corrected) was greater under Stable than under Volatile conditions (displayed at p<0.001, uncorrected). *N*=28.

**Figure S5**

*Learning Rate vs Discount Rate in Experiment 3*
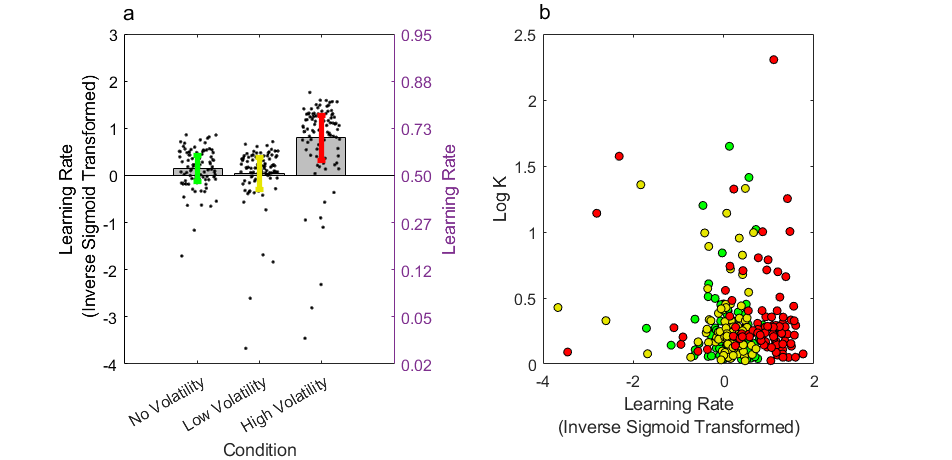


*Note.* a) Learning rate across the three conditions. Filled grey bars show group means, solid coloured bars 95% CIs; black dots show individual parameter estimates. b) Log K plotted against learning rate across conditions. No between-participant correlations (within condition) reached significance at p<0.05.

**Supporting Tables**

**Supporting Table 1**. Regions with significant activation for main parametric effect of Discounted Value, in a GLM without effects of condition, thresholded at p<0.001 uncorrected, at least 10 contiguous voxels.

| **Location** | **MNI coordinates** | ***T* Statistic** |
| --- | --- | --- |
| *R Dorsolateral Prefrontal Cortex*  *(Middle Frontal Gyrus)* | 42 8 40^1^  51 26 25^2^  48 38 -8 | 8.49    7.05  5.75 |
| *L Dorsolateral Prefrontal Cortex*  *(Middle Frontal Gyrus)* | -39 14 31  -48 14 28  -54 20 22 | 6.25  6.22  6.07 |
| *L Superior Temporal Gyrus* | -48 -46 10  -45 -37 -5  -63 -55 4 | 5.69  5.41  5.06 |
| *L Caudate* | -9 5 10  -15 -1 19 | 5.31  3.92 |
| *L Superior Parietal Cortex*  *(Angular Gyrus)* | -36 -55 40  -39 -43 40  -33 -64 52 | 5.30  4.37  3.95 |
| *R Superior Parietal Cortex*  *(Angular Gyrus)* | 42 -70 37  33 -55 43  39 -64 31 | 5.05  4.66  4.45 |
| *L Ventrolateral Prefrontal Cortex* | -39 50 5  -60 17 -5  -51 41 -2 | 4.96  4.94  4.70 |
| *L Anterior Insula* | -33 26 -2 | 4.46 |
| *L Precuneus* | -6 -64 43  -12 -67 58 | 4.42  4.39 |
| *L Anterolateral Prefrontal Cortex* | -27 65 4 | 4.41 |
| *R Middle Temporal Gyrus* | 63 -43 -2 | 4.40 |
| *R Midbrain* | 9 -19 -23 | 4.36 |
| *R Caudate* | 9 -1 13  12 2 4 | 4.07  3.89 |

1 FWE whole brain corrected, p<0.001

2 FWE whole brain corrected, p=0.007

**Supporting Table 2**. Regions with significant activation for the contrast Condition [Stable – Volatile] x Discounted Value, thresholded at p<0.001 uncorrected, at least 10 contiguous voxels.

| **Location** | **MNI coordinates** | ***T* Statistic** |
| --- | --- | --- |
| *L Temporal Pole* | -30 5 -26 | 4.35 |
| *L Anterior Insula* | -33 26 10 | 4.31 |
| *R Temporal Pole* | 33 8 -26 | 4.05 |

**Supporting Table 3**. Regions with significant increased coupling with L Hippocampus as a function of Condition [Stable – Volatile], within the volume of a mask for regions representing a parametric effect of discounted value (at p<0.001; as detailed in Supporting Table 1).

| **Location** | **MNI coordinates** | ***T* Statistic** |
| --- | --- | --- |
| *L Dorsolateral Prefrontal Cortex* | -39 26 25^1^  -42 26 34^2^ | 5.22  4.62 |

1 p=0.025 FWE corrected for search volume

2 p=0.090 FWE corrected for search volume

**Supporting Table 4**. Intertemporal choice set used for Experiment 2

| **Guaranteed immediate reward (£)** | **Delay (Weeks)** | **Delay (Days)** | **Simple hyperbolic *K* at indifference** |
| --- | --- | --- | --- |
| 24.88 | 0 | 0 | Inf |
| 24.72 | 0 | 0 | Inf |
| 24.38 | 0 | 0 | Inf |
| 23.65 | 0 | 0 | Inf |
| 22.16 | 0 | 0 | Inf |
| 19.41 | 0 | 0 | Inf |
| 15.16 | 0 | 0 | Inf |
| 10.16 | 0 | 0 | Inf |
| 24.88 | 1 | 7 | 0.0007 |
| 24.81 | 1 | 7 | 0.0011 |
| 24.72 | 1 | 7 | 0.0016 |
| 24.59 | 1 | 7 | 0.0024 |
| 24.38 | 1 | 7 | 0.0036 |
| 24.09 | 1 | 7 | 0.0054 |
| 23.65 | 1 | 7 | 0.0082 |
| 23.03 | 1 | 7 | 0.0122 |
| 22.16 | 1 | 7 | 0.0183 |
| 20.97 | 1 | 7 | 0.0275 |
| 19.41 | 1 | 7 | 0.0411 |
| 17.45 | 1 | 7 | 0.0618 |
| 15.16 | 1 | 7 | 0.0927 |
| 12.67 | 1 | 7 | 0.1390 |
| 10.16 | 1 | 7 | 0.2087 |
| 7.84 | 1 | 7 | 0.3127 |
| 24.88 | 3 | 21 | 0.0002 |
| 24.81 | 3 | 21 | 0.0004 |
| 24.72 | 3 | 21 | 0.0005 |
| 24.59 | 3 | 21 | 0.0008 |
| 24.38 | 3 | 21 | 0.0012 |
| 24.09 | 3 | 21 | 0.0018 |
| 23.65 | 3 | 21 | 0.0027 |
| 23.03 | 3 | 21 | 0.0041 |
| 22.16 | 3 | 21 | 0.0061 |
| 20.97 | 3 | 21 | 0.0092 |
| 19.41 | 3 | 21 | 0.0137 |
| 17.45 | 3 | 21 | 0.0206 |
| 15.16 | 3 | 21 | 0.0309 |
| 12.67 | 3 | 21 | 0.0463 |
| 10.16 | 3 | 21 | 0.0696 |
| 7.84 | 3 | 21 | 0.1042 |
| 24.88 | 8 | 56 | 0.0001 |
| 24.81 | 8 | 56 | 0.0001 |
| 24.72 | 8 | 56 | 0.0002 |
| 24.59 | 8 | 56 | 0.0003 |
| 24.38 | 8 | 56 | 0.0005 |
| 24.09 | 8 | 56 | 0.0007 |
| 23.65 | 8 | 56 | 0.0010 |
| 23.03 | 8 | 56 | 0.0015 |
| 22.16 | 8 | 56 | 0.0023 |
| 20.97 | 8 | 56 | 0.0034 |
| 19.41 | 8 | 56 | 0.0051 |
| 17.45 | 8 | 56 | 0.0077 |
| 15.16 | 8 | 56 | 0.0116 |
| 12.67 | 8 | 56 | 0.0174 |
| 10.16 | 8 | 56 | 0.0261 |
| 7.84 | 8 | 56 | 0.0391 |
| 24.88 | 17 | 119 | 0.0000 |
| 24.81 | 17 | 119 | 0.0001 |
| 24.72 | 17 | 119 | 0.0001 |
| 24.59 | 17 | 119 | 0.0001 |
| 24.38 | 17 | 119 | 0.0002 |
| 24.09 | 17 | 119 | 0.0003 |
| 23.65 | 17 | 119 | 0.0005 |
| 23.03 | 17 | 119 | 0.0007 |
| 22.16 | 17 | 119 | 0.0011 |
|  |  |  |  |
| 20.97 | 17 | 119 | 0.0016 |
| 19.41 | 17 | 119 | 0.0024 |
| 17.45 | 17 | 119 | 0.0036 |
| 15.16 | 17 | 119 | 0.0055 |
| 12.67 | 17 | 119 | 0.0082 |
| 10.16 | 17 | 119 | 0.0123 |
| 7.84 | 17 | 119 | 0.0184 |

*Note.* Hyperbolic K at indifference is calculated using a simple hyperbola, with a delayed reward magnitude of £25, and delay expressed in days. This same set of 72 choices was displayed once for the Stable product, and once for the Volatile product, organised in mini-blocks whose order was randomized.
